# Simultaneous abrupt shifts in hydrology and fish assemblage structure in a floodplain lake in the central Amazon

**DOI:** 10.1038/srep40170

**Published:** 2017-01-10

**Authors:** Cristhiana P. Röpke, Sidinéia Amadio, Jansen Zuanon, Efrem J. G. Ferreira, Cláudia Pereira de Deus, Tiago H. S. Pires, Kirk O. Winemiller

**Affiliations:** 1 Programa de Pós-Graduação em Biologia de Água Doce e Pesca Interior, Instituto Nacional de Pesquisas da Amazônia, Manaus, Amazonas, 69067-375, Brasil; 2 Faculdade de Ciências Agrárias, Universidade Federal do Amazonas, Manaus, Amazonas, 69077-000, Brasil; 3 Coordenação de Biodiversidade, Instituto Nacional de Pesquisas da Amazônia, Manaus, Amazonas, 69067-375, Brasil; 4 Department of Wildlife and Fisheries Sciences, Texas A&M University, College Station, TX, 77843-2258, USA

## Abstract

Combined effects of climate change and deforestation have altered precipitation patterns in the Amazon. This has led to changes in the frequency of extreme events of flood and drought in recent decades and in the magnitude of the annual flood pulse, a phenomenon that influences virtually all aspects of river-floodplain ecosystem dynamics. Analysis of long-term data revealed abrupt and synchronous changes in hydrology and fish assemblage structure of a floodplain lake near the confluence of Amazon and Negro rivers. After an intense drought in 2005, the assemblage assumed a different and fairly persistent taxonomic composition and functional structure. Declines in abundance after 2005 were more pronounced for species of all sizes having equilibrium life history strategy, large species with periodic life history strategy, and for all trophic levels except primary consumers. Our results suggest that the extreme drought triggered changes in the fish assemblage and subsequent anomalous hydrological conditions have hampered assemblage recovery. These findings stress the need to account for climatic-driven hydrological changes in conservation efforts addressing aquatic biodiversity and fishery resources in the central Amazon.

Climate change is impacting biodiversity and ecosystem services on a global scale^1^, however, consequences are expected to vary among regions and taxonomic and functional groups of organisms^2^. Most climate models predict that the tropics will experience large-scale changes in precipitation^3^, including regional increases in drought frequency and intensity^4^,^5^. Changes in precipitation patterns and frequency of extreme climatic events in the Amazon have become evident over recent decades, with reduced rainfall during the annual dry season in eastern and southern portions of the basin, and greater rainfall during the wet season in the western portion^6^,^7^. Recent extreme climatic events in the Amazon, such as the droughts of 1997, 2005 and 2010, and great floods of 2009, 2012, 2013 and 2014, have been related to greater Sea Surface Temperature anomalies in both Atlantic and Pacific oceans that change the water cycle in different parts of the basin and periods of the year^7^,^8^,^9^,^10^. The ongoing large-scale deforestation in the southern portion of the basin also may contribute to more extreme climatic events^11^,^12^,^13^,^14^.

Hydrology in the middle reaches of the Amazon is regulated by precipitation in catchments that span a vast portion of the basin^6^,^7^. Recent changes in the distribution of precipitation among sub-basins have intensified the amplitude and duration of annual dry and wet phases of the flood pulse in middle and lower reaches of the Amazon^6^,^15^, changing seasonal factors that affect aquatic organisms living in these areas. Similar to how temperature and day length regulate primary productivity, resource dynamics and other ecological factors in temperate ecosystems, water level fluctuation is responsible for ecological dynamics and environmental quality (e.g. dissolved oxygen and nitrogenous compounds in water) in floodplain lakes of the Amazon Basin^16^,^17^. Fish provide one of the clearest examples of ecological responses to seasonal variation of hydrology in the Amazon. During the flood season, greater availability of aquatic habitat and food resources enhances feeding opportunities, early life-stage survival, and storage of fat that helps sustain fish throughout the dry season when many resources become scarce and most fish populations experience greater competition and predation mortality^16^. Given that changes in the timing, magnitude and duration of phases of the annual flood pulses affect local fish populations, they also should influence species assemblage structure.

Consequences of hydrological changes on the Amazonian fish fauna are poorly understood. Only a few studies have examined impacts of recent climatic change on aquatic ecology in the Amazon, and these have analyzed responses to drought over relatively short time intervals^18^,^19^. The Amazon has the most diverse freshwater fish fauna on Earth^20^, with the highest functional diversity^21^. High functional diversity could enhance or reduce community resilience to disturbance, depending on whether functional redundancy is high^22^, or if many species are ecological specialists with limited tolerance to disturbances^23^. However, resilience depends heavily on the magnitude of environmental disturbance, and species may respond differently, with certain traits either facilitating or hindering population resilience^24^. Given concerns for loss of biodiversity and ecosystem services in response to human actions^1^, there is a pressing need for long-term monitoring of biological communities and ecosystems in order to understand factors affecting their vulnerabilities, resistance and resilience to environmental change.

Here we investigate temporal changes in fish assemblage structure during the period of 1999–2014, when the central Amazon experienced abnormally large inter-annual hydrological variation. To evaluate trends in inter-annual and intra-annual hydrologic variation, we analyzed river discharge data from 1950 to 2014 using principal components analysis (PCA). Multivariate ordination methods also were used to assess taxonomic and functional assemblage structures, the latter according to two ecological aspects: life-history strategies and trophic positions. Fish abundance data were obtained from standardized fish surveys conducted in a floodplain lake (Lago Catalão) near the confluence of Negro and Amazon rivers (Fig. 1a, study area description is presented in the supplementary Material and Methods). We applied a statistical procedure^25^ that detects abrupt shifts and correspondence in paired time-series data (i.e., hydrology vs. fish assemblage structure), estimates response functions of shifts, and indicates whether shifts resulted from external or internal drivers.

## Results

**Figure 1:**
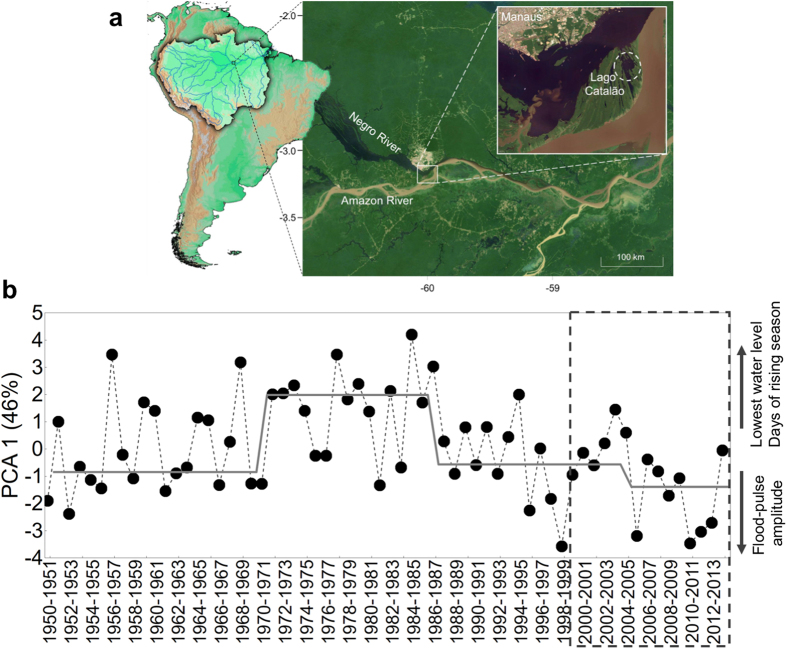
Map showing location of the study system and graph showing hydrological variation from 1950 to 2014. (**a**) Lago Catalão location at the confluence of Amazon and Negro rivers, and the city of Manaus, Brazil (Map data: inner close-up image Google, DigitalGlobe; outer image Google, Landsat). The left image shows the position in relation to Amazon Basin and South America (map generated using QGIS v2.14, http://qgis.org/en/site/). (**b**) Temporal variation in hydrology as indicated by scores on the first axis from PCA using seven hydrologic variables (see Material and Methods, Dataset S1). Mean PCA1 values are plotted as a continuous grey line. Period of fish surveys is highlighted by the dashed rectangle.

Multivariate ordination of hydrological data produced a dominant gradient in the first axis (PCA1) that contrasted annual flood pulses with lower amplitudes, dry seasons with higher water levels, and longer periods of rising water level with the opposite set of conditions (Fig. 1b). In the first axis, three significant moments of change were identified in the hydrological variation over the last 65 years (1971, 1987 and 2005; Fig. 1b; Supplementary Table S1). After the significant hydrological change in 2005–2006, annual flood pulses tended to have higher amplitudes and water levels during the dry season were lower (Fig. 1b). The second axis of the multivariate ordination (PCA2) had significant hydrological changes in 1957, 1962, 1969, 1979, 1986 which contrasted conditions associated with shorter dry seasons or shorter wet seasons (see Supplementary Fig. S1).

**Figure 2:**
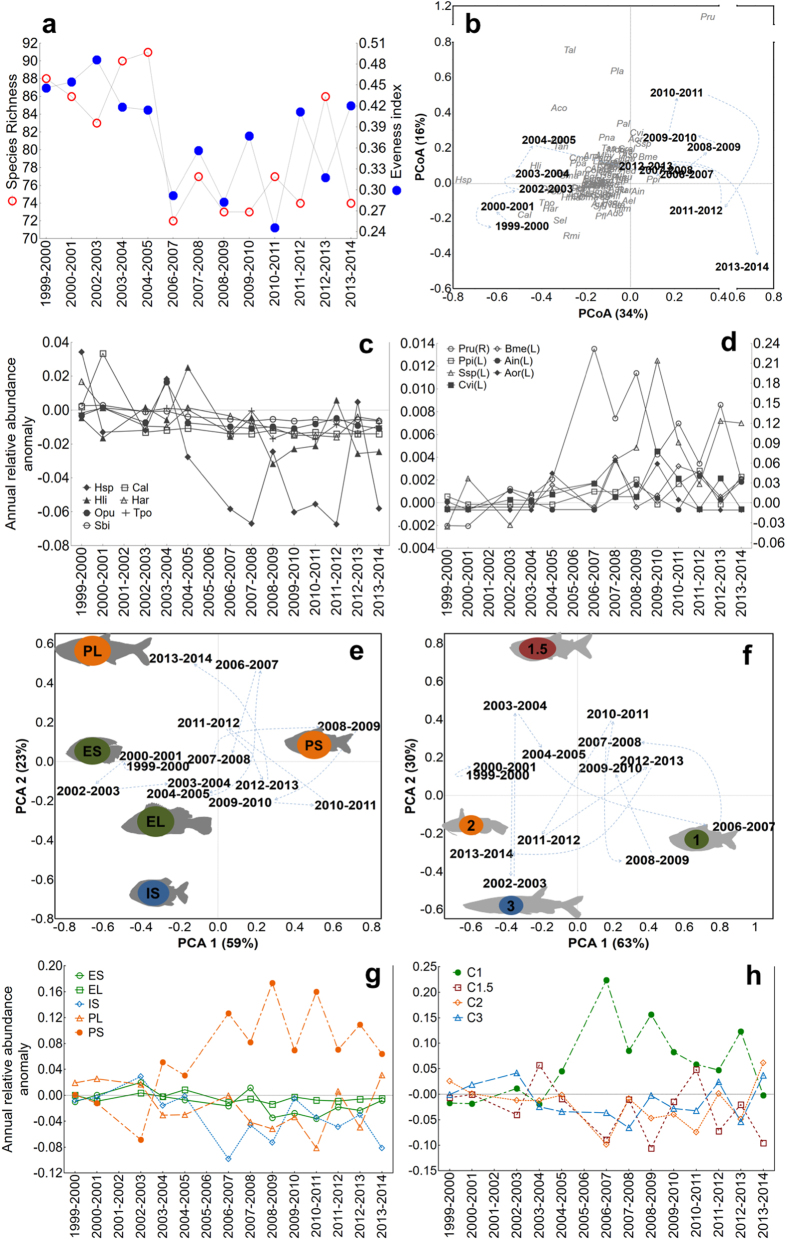
Temporal variation in species diversity and evenness, and ordination plots showing temporal variation in assemblage structure. (**a**) Temporal variation in diversity (measured by species richness) and species evenness of the fish assemblage of Lago Catalão; (**b**) ordination plot revealing temporal variation in taxonomic structure, based on analysis of 97 species with abundance >0.01 individuals/m^2^ (CPUE); positive scores on PCoA1 are strongly associated with greater abundance of *Psectrogaster rutiloides* (Pru), *Pinirampus pirinampu* (Ppi) and *Brycon melanopterus* (Bme), and negative scores associated with greater abundance of most other species (species abbreviations appear in Dataset S2); (**c**) anomaly of the relative abundance for the seven species with lowest scores which decreased in abundance over time, and (**d**) for the seven species with highest scores which increased in abundance over time; values for species Pru are presented in the second y axis because its abundance is much greater than other species. (**e**) Ordination plot revealing temporal variation in life history structure: equilibrium-small (ES), equilibrium-large (EL), intermediate-small (IS), periodic-large (PL) and periodic-small (PS). (**f**) Ordination plot revealing temporal variation in trophic structure (C1- consumer level 1; C1.5- consumer level 1.5; C2- consumer level 2; C3- consumer level 3). Light blue dashed lines connect sequential years; vector scores were multiplied by a constant to improve plot visualization. (**g**) anomaly of the relative abundance for each life history group and (**h**) trophic level in the time series. Life history strategies based on data from specimens collected during surveys and review of published information and criteria in Winemiller (1989), and vertical trophic positions based on primary data and review of published dietary information. (**c**,**d**,**g**,**h**) Anomaly was calculated as the annual relative abundance minus the mean relative abundance before 2005, note that y axes of plots are scaled differently.

**Figure 3:**
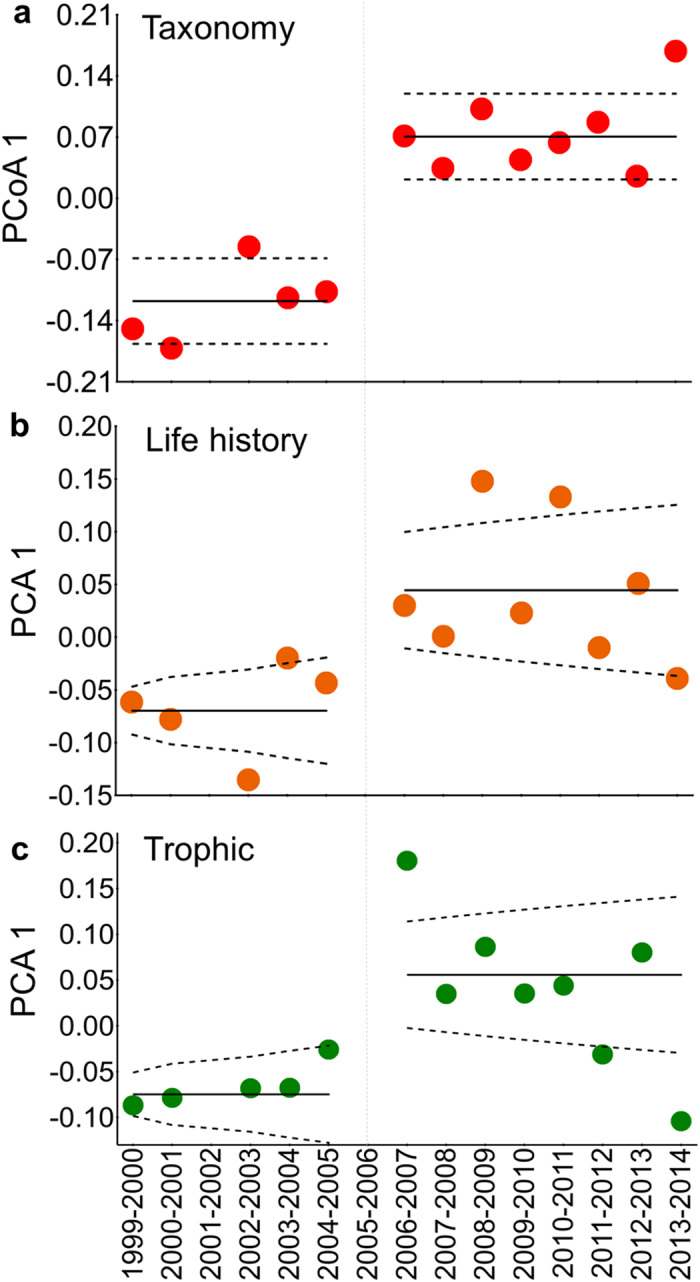
Temporal dynamics of assemblage structure as defined by scores on the first axis from PCA or PCoA ordinations. Solid lines represent best-fit regression models describing time-series data (Table S2); dashed lines represent confidence intervals. Segmented step-mean regressions for each of three aspects of assemblage structure indicate major shifts following the 2005 drought followed by persistent structures under a new hydrologic regime (Fig. 1b).

Taxonomic and functional composition of the fish assemblage differed markedly before and after 2005 (Fig. 2); the relative abundance of some species increased and overal fish diversity declined (Fig. 2a–d). Relative abundances of periodic-small (PS) and first-level consumers increased in the assemblage, while species with other life history strategies and trophic positions tended to have reduced relative abundances (Fig. 2e–h). Major shifts in both taxonomic and functional assemblage structures in 2005 were indicated by changes in scores on the dominant axis from multivariate analysis (Supplementary Table S1), and these shifts conformed to a segmented step-mean model (Fig. 3, Table S2). Simulations were performed to evaluate the effect of the missing value for the flood pulse cycle of 2005–2006 in the PCA 1 (Supplementary Tables S3–S5). Nearly all simulations also produced a segmented step-mean as the best-fit model for taxonomic and life-history structures (98% and 100% of simulations for taxonomy and life history, respectively). The best-fit model (74% of simulations) for changes in assemblage trophic structure was a segmented linear-linear model, with positive-negative slopes. Life history and trophic structures of the fish assemblage showed greater variation after 2005 (Fig. 3b,c). During this later period, several years having similar hydrological conditions (i.e. 2006–2007, 2007–2008, 2008–2009 and 2010–2011, 2011–2012, 2012–2013) had assemblages with dissimilar functional structures (Fig. 3). A null model yielded the best fit for temporal variation in taxonomic assemblage structure as described by PCA axis 2 (Supplementary Fig. S2, Table S2), indicating no trend and implying that the first axis had explained most of the temporal variation in assemblage structure.

**Figure 4:**
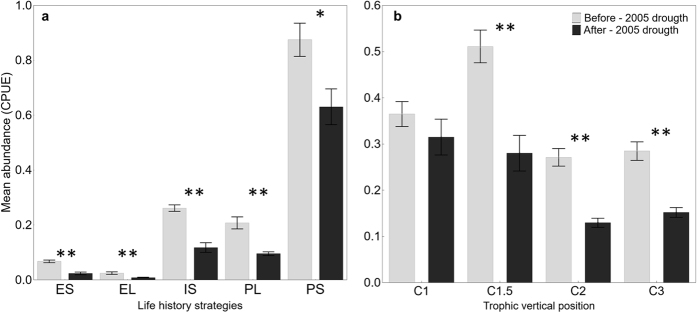
Mean abundance of functional groups before and after the 2005 drought; mean and SE according to life history (**a**) and vertical trophic position (**b**). Note that y axes of plots are scaled differently; asterisks indicate significant differences in group abundance between the two periods (*P < 0.05; **P < 0.001). Trophic and life history strategy abbreviations are shown in Fig. 2.

The 2005 drought significantly affected abundance (capture per unity effort CPUE) of most groups (life history: RM-ANOVA F _5,7_ = 13.54, *P* = 0.001; trophic levels: RM-ANOVA F _4,8_ = 14.527, *P *< 0.001; Fig. 4; details appear in Supplementary Table S6). In contrast to changes that occurred following the 2005 drought, mean abundance (CPUE) of the functional groups did not decline or increase significantly during the five years following the 2010 drought (details are shown in Supplementary Table S7). Although this suggests no effect of the more recent 2010 drought on fish abundance patterns, this extreme event may have contributed to the failure of assemblage to return to its pre-2005 structure.

**Figure 5:**
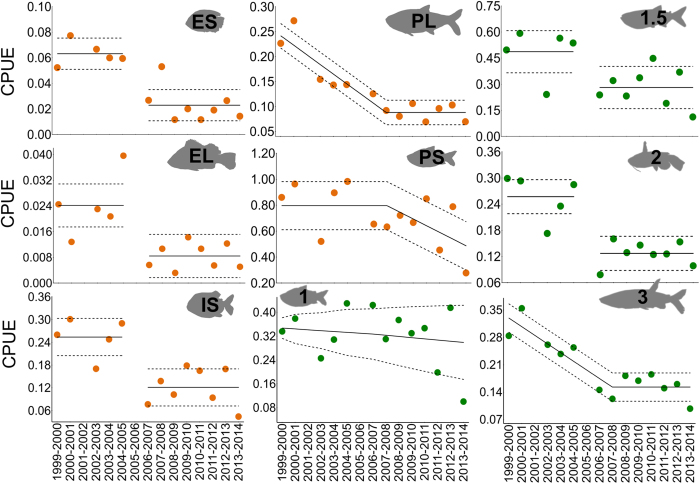
Temporal trends in abundance (CPUE) of functional groups identified by regression analysis. Solid lines represent best-fit model predictions; dashed lines represent standard errors around mean estimates (Table S2). Abrupt shifts occurred after 2005 for ES, EL and IS life history strategies, and omnivores (consumer trophic level 1.5) and carnivores (consumer trophic level 2). Temporal changes were more gradual but with inflections during 2007–2008 for PL and PS life history strategies and piscivores (consumer trophic level 3). Abundance of herbivores and detritivores (consumer trophic level 1) revealed a trend of gradual decline but with poor model fit. Datasets S4–S5. Note that y axes of plots are scaled differently. Trophic and life history strategy abbreviations and symbols are shown in Fig. 2.

Considering temporal trends of abundance, as CPUE values, of each functional group over the studied period, a segmented step-mean model was the best fit for abundance dynamics of ES, EL, IS strategists, and trophic levels 1.5 (omnivores) and 2 (secondary consumers), all of which shifted to lower values after 2005 and then remained relatively stable (Fig. 5). However, a segmented negative-linear, stable-mean model produced the best fit for abundance of PL strategists and tertiary consumers (piscivores), with the transition occurring in 2008. Best fit for abundance of PS strategist was a stable-mean until 2008, followed by a negative linear trend, suggesting delayed response. The best-fit model for primary consumers (after correcting for variance heterogeneity) was a monotonic decline with shallow non-significant slope (R^2^ = 0.19, *P* = 0.13, Fig. 5).

## Discussion

The year of 2005 corresponded to the first extreme drought that occurred within our field study time frame and after which a set of changes occurred in the river hydrological regime^10^. Coincident with these hydrological changes, the fish assemblage structure of Lago Catalão changed abruptly after 2005, and the new structure was maintained over the following decade. A study conducted from 2004–2007 on six floodplain lakes^18^ located upstream from our study area also documented changes in taxonomic and trophic structure of fish assemblages after 2005. Thus, effects of drought on fish assemblages in the central Amazon have been revealed by two independent studies, which suggests that environmental changes affecting fish ecology may have occurred at a regional scale. Local environmental conditions and fish assemblage structure vary among floodplain lakes as a consequence of hydrological connectivity^26^,^27^,^28^ and water quality^29^ among other factors. To determine the potential for climate change to induce shifts in hydrology and fish assemblage structure over a broad regional scale, long-term, standardized monitoring and analyses are needed for other regions of the Amazon. Similar to the results reported here, Freitas and coworkers^18^ also found that the 2005 drought was associated with a reduction in the relative abundance of fish species with an equilibrium-type life history strategy, with a lesser response by periodic strategists. Lower relative abundances of equilibrium life-history strategists in systems with large intra-annual variation in water level have been reported for fish assemblages of other tropical rivers as well as fluvial systems in other regions of the world^30^,^31^,^32^.

Significant changes in Amazon floodplain fish assemblages after the 2005 drought have now been reported by two different studies, this supports the hypothesis that recent extreme climatic events have altered key environmental factors affecting fish ecology. The Amazon has undergone unprecedented shifts in rainfall and hydrology over the last 10–12 years^10^,^11^,^14^. More extreme droughts and floods have occurred after 2005, and appear to be associated with large-scale climatic events, including El Niño, La Niña and Atlantic Sea Surface Temperature changes, and their interactions^8^,^10^. In addition, deforestation in the Amazon may have a synergistic effect with large-scale atmospheric circulation anomalies that impacts precipitation patterns and Amazon River hydrology^6^,^10^.

The similarity of response functions obtained for hydrological and biological time series, together with concordance of their change points and the fundamental importance of seasonal flood pulsing for fish ecology, strongly implies that hydrology (which in turn mediates the quality of aquatic habitat) was the main driver (*sensu* ref. 33) of the observed structural changes in the Lago Catalão fish assemblage. The environmental perturbation caused by the extreme drought of 2005, which dried out about 70% of aquatic habitats in the Amazon floodplains^34^, is the most plausible trigger for the abrupt change in assemblage structure. Several mechanisms probably account for synchronicity of changes in assemblage structural components, including 1) migration restriction and 2) increased mortality due to harsh abiotic conditions and predation, owing to higher per-unit-area fish densities within smaller, less-connected aquatic habitats during the dry season^35^,^36^. However, not every functional change was synchronous, which suggests a significant influence from intrinsic biological factors, or additional environmental drivers that were not analyzed here. For example, the relatively stable, although lower, abundance of EL and ES strategists after 2005, might be explained by their capacity for density compensation in response to biotic factors^37^, an attribute that may have counterbalanced their relatively weak demographic resilience in response to large-scale abiotic environmental variation (e.g., greater amplitude of flood pulses after 2005, Fig. 1b). The decline in abundance of PS strategists after 2008 probably was influenced by reduced lateral connectivity during dry seasons. Several of these species migrate seasonally between floodplain lakes and the river channel for feeding and reproduction (e.g., *Hemiodus* sp., *Curimatella alburna, Triportheus angulatus*), and would be especially affected by reduced habitat connectivity during longer dry seasons^35^. Mean abundance of PL strategists declined gradually until 2008, after which abundance was stable at a lower level. This group contains many wide-ranging migratory species that typically spawn during the beginning of the ascending phase of the annual flood pulse (e.g., *Brycon* spp., *Colossoma macropomum, Prochilodus nigricans, Semaprochilodus* spp.), and therefore should be particularly sensitive to changes in flood-pulse timing as well as lateral connectivity.

Temporal variation in the abundance of various trophic groups should be linked to the strong influence of hydrology on aquatic primary production, availability of terrestrial food resources, and habitat availability during the dry season that influences fish density and predation rates^38^. The relatively small changes in abundance of primary consumers probably can be explained by minimal changes in food availability for these fishes, most of which can shift between feeding on algae, detritus and, in some cases, aquatic or riparian plants^39^. Mean abundance of omnivores and secondary consumers declined after 2005, and these large and diverse groups could have been affected by changes in predation as well as food resources. Earlier onset of flood recession and longer periods of drought should have resulted in higher fish densities and lower access to habitats that serve as predation refuges, factors that also could account for the comparatively stable abundances of many piscivores after 2008.

Our analyses suggest that recent climatic and hydrological changes have altered aquatic ecology of the Lago Catalão. Species diversity declined and multiple aspects of fish assemblage structure changed after the strong drought of 2005. Lower diversity with greater temporal variability is consistent with the hypothesis that loss of biodiversity reduces community stability^40^. Our findings reinforce the importance of hydrology as the major extrinsic driver of assemblage structure of fishes inhabiting floodplain habitats. In addition, fishing pressure^41^ and deforestation^42^ in the central Amazon could have contributed to reduced resilience of certain fish stocks (e.g., large species with relatively long generation times, represented in our study by EL and PL life history strategies) to hydrological changes. Because central Amazon fisheries preferentially target large species^43^, it will be difficult to quantify effects of hydrology and fishing on fish populations and assemblages separately. Continuous monitoring and analysis of longer time series are needed to disentangle effects caused by more frequent occurrence of extreme drought, persistent changes in hydrology, fisheries and other extrinsic as well as intrinsic drivers of fish assemblage structure. This would allow the assessment of whether the observed changes are stable or represent a transitory state. In order to assess the potential impact of climate change on aquatic ecology in the Amazon, more research is needed to produce long time series for other floodplain lakes and aquatic habitats. Analysis of more extensive time-series data should promote the construction of predictive models to guide conservation of Amazonian aquatic biodiversity and fisheries. This assessment will be critical not only for biodiversity conservation, but also for fisheries management and food security for people in the central Amazon.

## Methods

### Fish surveys and ecological information

Fishes have been surveyed monthly since October 1999, using a standardized method, in Lago Catalão, near Manaus (Fig. 1a). Lago Catalão is a floodplain lake with spatially and temporally heterogeneous water chemistry due to the direct influence of both the Amazon and Negro rivers during flood pulse cycles (Fig. 1a, see the supplementary Material and Methods for a detailed description of the study area). For the present investigation, we analyzed data of surveys from October 1999 until October 2014. The dataset has two gaps caused by logistics problems that resulted in incomplete sampling (October 2001 to September 2002 and October 2005 to November 2006) and because of that were not included in the study. Fishes were sampled using sets of 10 gillnets with multiple mesh sizes (details appear in ref. 35 and supplementary Material and Methods). In the laboratory, each specimen was identified, measured for standard length (SL, mm), weighed (g) and examined to determine sex and reproductive status according to criteria described in ref. 44. Dietary analysis was based on frequency of occurrence and relative volume of food items in stomachs (detailed methods in ref. 45).

### Hydrology – annual flood-pulse attributes

Daily records of the water level in the Negro River in Manaus were obtained from the website of Porto de Manaus Company (http://www.portodemanaus.com.br) and Brazil’s National Agency for Water (http://hidroweb.ana.gov.br). Temporal components of the annual flood pulse were defined based on analysis of water level recorded over 100 years^46^. Dry season was defined as the period when the stage level is lower than 20 m; the rising season was when the water level is between 20 m and 26 m with an ascending vector (increasing water level); flood season was when the stage level is higher than 26 m; and the receding season was when the stage level is between 26 m and 20 m with a descending vector (lowering water level)^46^. Each annual flood pulse was defined as the, onset of the dry season of a given year and the dry season of the subsequent year. Specifically, the time interval from when the ascending water level was at 20 m until it had peaked and declined again to 20 m. Seven variables were used to describe hydrological dynamics: amplitude of the flood pulse; annual lowest water level (stage level); annual highest water level; number of days of the dry season; number of days of the rising season; number of days of the flood season; and number of days of the receding season (Dataset S1). Principal components analysis (PCA) was used to ordinate years according to hydrological variables that changed over the last 65 years (since 1950). Prior to analysis, data were standardized (see the supplementary Material and Methods for a detailed description); PCA was performed using *stats* library and *prcomp* function^47^ in R software^48^. The number of axes considered for interpretation and further analysis was delimited by Scree Plot and Broken Stick methods. For detection of changes on hydrological time series, the Sequential t-test Analysis of Regime-Shifts algorithm (STARS)^49^,^50^ was applied to ordination data (axis 1 and 2) from the PCA, which modeled 82% of total variation. Time-series data were passed through a white-noise filter using the ordinary least-squares method^49^,^50^. We set the significance level value at 0.05, and the window size to identify significant change points was set to 10 years.

### Fish assemblage structure

Data used to analyze assemblage taxonomic and functional structures and patterns of population abundance were organized according to annual flood pulse cycles (in effect, “years”) as defined for the hydrological analysis (see previous paragraph).

Taxonomic structure - In total, 97 common species out of 196 total species were included in the data matrix for analysis (details in supplementary Material and Methods). To avoid potential bias from inter-annual differences in sampling effort (e.g., differences in the total number of months to complete the flood pulse cycle, for instance the cycle of 2004–2005 lasted 10 months and 2002–2003 lasted 13 months, see Dataset S2) abundance data were transformed to catch per unit effort (CPUE) (Dataset S2). Similarities in assemblage composition among annual flood pulses were investigated by principal coordinates analysis (PCoA). Prior to analysis, data were square root transformed to reduce the influence of abundant species^51^ and converted into a Bray-Curtis dissimilarity distance matrix using the *vegdist* function in *vegan* library. PCoA analysis was performed using *stats* and *BiodiversityR*.

Life history - For all 97 species, we obtained information on five life history traits that were considered likely to influence how populations respond to patterns of environmental variation and allow assignment of species into life history strategies^30^,^52^,^53^. These traits were maximum size, size at sexual maturation, fecundity, oocyte diameter, and degree of parental care (Dataset S4). For detailed description on the life history traits measurements and supporting references, see supplementary Material and Methods. We performed PCA using life history data to ordinate species along a continuum of strategies^52^. From the resulting correlations between species traits and PC axis scores, we identified five groups among species ordinated by the fecundity and parental care gradients (PCA 1–50%), and secondarily by oocyte diameter, maximum size, and size at sexual maturation (PCA 2–28%) (Supplementary Fig. S3): *Equilibrium-small* (ES, 12 species); *Equilibrium-large* (EL, 3 species); *Intermediate-small* (IS, 18 species); *Periodic-large* (PL, 29 species); *Periodic-small* (PS, 35 species). Fishes with an opportunistic life history strategy (i.e., small size, early maturation, high and sustained reproductive effort but low batch fecundity and little or no parental care) essentially were missing from our samples, because they were too small (<60 mm SL) to be caught by the standardized sampling method (gillnets). Further details on criteria used to categorize species according to life-history traits can be found in supplementary Material and Methods.

Trophic level - Each species was assigned one of the four trophic levels: *Consumer trophic level 1*- herbivorous and detritivorous species (n = 26); *Consumer trophic level 1*.*5*- omnivorous species (n = 21); *Consumer trophic level 2*- invertivorous and planktivorous species (n = 26); *Consumer trophic level 3*- piscivorous species (n = 24) (Dataset S5). Further details on criteria used to categorize species can be found in supplementary Material and Methods.

Temporal variation in life history and trophic assemblage structures was assessed using PCA^25^,^51^. Data were Hellinger transformed using the *decostand* function in the *vegan* library to reduce influence of more abundant groups but keeping proportionality before performing PCA^54^. The number of axes retained for subsequent analysis was determined based on Scree Plot and Broken Stick methods.

## Statistical Approaches

### Drought effect on abundance of functional groups

The effect of 2005 and 2010 droughts on abundance (CPUE) of each functional group was tested by Repeated-measures analysis of variance (RM-ANOVA) using two analytical designs: i) years before 2005 compared to years after 2005; ii) years after 2005 drought (2006–2007, 2007–2008, 2008–2009, 2009–2010) compared to after 2010 drought (2010–2011, 2011–2012, 2012–2013, 2013–2014). Assumptions regarding data normality and homoscedasticity were met for this analysis.

### Temporal changes in fish assemblage and functional groups abundance trends

To evaluate temporal changes of fish assemblage (PCA and PCoA object scores) and temporal trends of abundance (CPUE) of each functional group, we applied a statistical procedure recently proposed to identify regime shifts in time series data^25^,^55^. This procedure consists of testing for the best-fit models describing response functions (types of trends) and significance of shift moments. Change moment was identified using the STARS algorithm^49^,^50^. The biotic time-series data were passed through a white-noise filter using the ordinary least-squares method^49^,^50^. We set the significance level value to 0.01. Cut-off lengths for the window size to identify moment (year) of significant change were tested using two different values, 7 and 10 years, which represented a long and a short time window. Both values resulted in the same regime shift moment (2006–2007). The STARS indicated 2006–2007 (our first-year sampling after the strong drought of 2005, Table S1) as the most frequent shift moment, this moment was set in the regressions to test the possible trend changes defined in the response functions.

Response functions and significance of shifts were tested using generalized nonlinear least-squares regression, a method that allows dealing with nonlinear relationships and non-normal errors^25^,^56^. We tested the relationship between response and years by fitting six different models which could test multiple hypotheses to explain trajectories^25^: null, linear, segmented linear-linear, segmented step-mean, segmented linear – stable mean, segmented stable mean – linear, and sigmoidal relationships, using the *nlme* package and *gnls* function^57^, based on published equations^25^. The best model was identified using the Akaike Information Criterion with a correction for finite sample sizes (AICc), using *AICcmodavg* library and *AICc* function^58^. Residuals of the best model were checked for temporal autocorrelation, and the trend was reanalyzed with the autocorrelation formulated as part of the covariance matrix of the observations. Autocorrelation and heterogeneity of variance were controlled by including in the model an autoregressive correlation term and variance term for error^25^,^56^. A new AICc was applied to select the best model. The effect of the missing data in 2005–2006 was evaluated by simulating values of PC1 for the year of 2005–2006. We generated all possible values from the lowest value of PC1 to its maximum, with increments of 0.001. Best-fit model analysis was conducted for each simulated value, and after the completion of all simulations, we calculated the proportion of the total runs that each model was retained as the best-fit (lowest AICc). This procedure was repeated for all assemblage structures (taxonomic and functional; Tables S3–S5). All analyses were performed with R software.

The possible driving factors of assemblage dynamics (i.e., the relative importance of intrinsic processes, such as species interactions, and extrinsic factors, such as environmental perturbation) were inferred by comparing the type of response function between environmental and assemblage structure changes^33^. Similar response functions were interpreted as indicative of extrinsically driven regime shifts whereby environmental change induces shifts in the fish assemblage. In contrast, non-corresponding response functions were interpreted as a consequence of internal drivers or other unmeasured factors.

## Additional Information

**How to cite this article**: Röpke, C. P. *et al*. Simultaneous abrupt shifts in hydrology and fish assemblage structure in a floodplain lake in the central Amazon. *Sci. Rep.* **7**, 40170; doi: 10.1038/srep40170 (2017).

**Publisher's note:** Springer Nature remains neutral with regard to jurisdictional claims in published maps and institutional affiliations.

## Supplementary Material

Supplementary Information

Supplementary Dataset 1

Supplementary Dataset 2

Supplementary Dataset 3

Supplementary Dataset 4

Supplementary Dataset 5

## References

[b1] Bellard C. . Impacts of climate change on the future biodiversity. Ecol. lett. 15, 365–377 (2012). 22257223 10.1111/j.1461-0248.2011.01736.x PMC3880584

[b2] Hansen G. & Cramer W. Global distribution of climate change impacts. Nature Clim. Change 5, 182–184 (2015).

[b3] Diffenbaugh N. S. & Giori F. Climate change hotspots in the CMIP5 global climate model ensemble. Clim. Change 114, 813–822 (2012). 24014154 10.1007/s10584-012-0570-x PMC3765072

[b4] Duffy P. B., Brando P., Asner G. P. & Field C. B. Projections of future meteorological drought and wet periods in the Amazon. Proc. Natl. Acad. Sci. USA 112, 13172–13177 (2015). 26460046 10.1073/pnas.1421010112 PMC4629378

[b5] Tremberth K. E. . Global warming and changes in drought. Nature Clim. Change 4, 17–22 (2013).

[b6] Gloor M. . Intensification of the Amazon hydrological cycle over the last two decades. Geophys. Res. Lett. 40, 1–5 (2013).

[b7] Tomasella J. . The droughts of 1997 and 2005 in Amazonia: floodplain hydrology and its potential ecological and human impacts. Clim. Change 116, 723–746 (2013).

[b8] Marengo J. A. . Hydro-climatic and ecological behaviour of the drought of Amazonia in 2005. Phil. Trans. R. Soc. Lon. B. 363, 1773–1778 (2008). 18270160 10.1098/rstb.2007.0015 PMC2373880

[b9] Sorribas M. V. . Projections of climate change effects on discharge and inundation in the Amazon basin. Clim. Change, doi: 10.1007/s10584-016-1640-2 (2016).

[b10] Marengo J. A. & Espinosa F. C. Extreme seasonal droughts and floods in Amazonia: causes, trends and impacts. Int. J. Climatol., doi: 10.1002/joc.4420 (2015).

[b11] Malhi Y. . Climate change, deforestation, and the fate of the Amazon. Science 319, 169–172 (2008). 18048654 10.1126/science.1146961

[b12] Bagley J. E. . Drought and Deforestation: has land cover change influenced recent precipitation extremes in the Amazon? J. Clim. 27, 345–361 (2014).

[b13] Coelho C. A. S. . Climate diagnostics of three major drought events in the amazon and illustrations of their seasonal precipitation predictions. Meteorol. Appl. 19, 237–255 (2012).

[b14] Borma L. de S., Nobre C. A. & Cardoso M. F. Response of the Amazon Tropical Forests to Deforestation, Climate, and Extremes, and the Occurrence of Drought and Fire In Climate Vulnerability: Understanding and Addressing Threats to Essential Resources (ed. Pielke R. A. Sr.) 153–163 (Elsevier Inc., Academic Press, 2013).

[b15] Satyamurty P., Costa C. P. W., Manzi A. O. & Candido L. A. A quick look at the 2012 record flood in the Amazon Basin. Geophys. Res. Lett. 40, 1396–1401 (2013).

[b16] Lowe-McConnell R. H. Ecological studies in tropical fish communities (Cambridge University Press, Cambridge) (1987).

[b17] Junk W. J. The Central Amazon Floodplain: Ecology of a Pulsing System (Ecological Studies, Springer Verlag, Berlin, Heidelberg and New York) (1997).

[b18] Freitas C. E. C., Siqueira-Souza F. K., Humston R. & Hurd L. E. An initial assessment of drought sensitivity in Amazonian fish communities. Hydrobiologia 705, 159–171 (2013).

[b19] Correia G. B., Siqueira-Souza F. K. & Freitas C. E. C. Intra- and inter-annual changes in the condition factors of three Curimatidae detritivores from Amazonian floodplain lakes. Biota Neotropica 15, 1–7 (2014).

[b20] Pimm S. L. . The biodiversity of species and their rates of extinction, distribution, and protection. Science 344, 1246752 (2014). 24876501 10.1126/science.1246752

[b21] Toussaint A., Charpin N., Brosse S. & Villéger S. Global functional diversity of freshwater fish is concentrated in the Neotropics while functional vulnerability is widespread. Sci. Rep. 6, 22125, doi: 10.1038/srep22125 (2016). 26980070 PMC4793233

[b22] Nash K. L. . Herbivore cross-scale redundancy supports response diversity and promotes coral reef resilience. J. Appl. Ecol. 53, 646–655 (2015).

[b23] Clavel J., Julliard R. & Devictor V. Worldwide decline of specialist species: toward a global functional homogenization? Front. Ecol. Environ. 9, 222–228 (2011).

[b24] Moritz C. & Agudo R. The future of the species under climate change: Resilience or decline? Science 341, 504–508 (2013). 23908228 10.1126/science.1237190

[b25] Seddon A. W. R., Froyd C. A., Witkowski A. & Willis K. J. A quantitative framework for analysis of regime shifts in a Galápagos coastal lagoon. Ecology 95, 3046–3055 (2014).

[b26] Pouilly M. & Rodríguez M. A. Determinism of fish assemblage structure in neotropical floodplain lakes: influence of internal and landscape lake conditions In Proceedings of the Second International Symposium on the Management of Large Rivers for Fisheries (LARS2) (eds Welcomme R. & Petr T.) 243–265 (FAO, RAP Publication, 2004).

[b27] Granado-Lorencio C., Araujo-Lima C. R. M. & Lobón-Cervia J. Abundance - distribution relationships in fish assembly of the Amazonas floodplain lakes. Ecography 28, 515–520 (2005).

[b28] Freitas C. E., Siqueira-Souza F. K., Florentino A. C. & Hurd L. E. The importance of spatial scales to analysis of fish diversity in Amazonian floodplain lakes and implications for conservation. Ecology of Freshwater Fish 23, 470–477 (2014).

[b29] Saint-Paul U. . Fish communities in central Amazonia white-and blackwater floodplains. Environmental Biology of Fishes 57, 235–250 (2000).

[b30] Tedesco P. A. . River hydrological seasonality influences life history strategies of tropical riverine fishes. Oecologia 156, 691–702 (2008). 18368426 10.1007/s00442-008-1021-2

[b31] Mims M. C. & Olden J. D. Life history theory predicts fish assemblage response to hydrologic regimes. Ecology 93, 35–45 (2012). 22486085 10.1890/11-0370.1

[b32] Olden J. D. & Kennard M. J. Intercontinental comparison of fish life history strategies along a gradient of hydrologic variability In Community ecology of stream fishes: concepts, approaches, and techniques (eds. Gido K. B. & Jackson D. A.) 83–107 (American Fisheries Society, 2010).

[b33] Williams J. W., Blois J. L. & Shuman B. N. Extrinsic and intrinsic forcing of abrupt ecological change: case studies from the late Quaternary. J. Ecology 99, 664–677 (2011).

[b34] Frappart F. . Surface freshwater storage and dynamics in the Amazon basin during the 2005 exceptional drought. Environ. Res. Lett. 7, 044010 (2012).

[b35] Röpke C. P., Amadio S., Winemiller K. O. & Zuanon J. Seasonal dynamics of the fish assemblage in a floodplain lake at the confluence of the Negro and Amazon Rivers. J. Fish Biol. 89, 194–212 (2016). 26563716 10.1111/jfb.12791

[b36] Legder M. E. . Drought alters the structure and functioning of complex food webs. Nature Clim. Change 3, 223–227 (2012).

[b37] Rose K. A. . Compensatory density dependence in fish populations: importance, controversy, understanding and prognosis. Fish Fish. 2, 293–327 (2001).

[b38] Ledger M. E. . Impact of simulated drought on ecosystem biomass production: an experimental test in stream mesocosms. Glob. Change Biol. 17, 2288–2297 (2011).

[b39] Mortillaro J. M. . Trophic opportunism of central Amazon floodplain fish. Freshwater Biol. 60, 1659–1670 (2015).

[b40] Hautier Y. . Anthropogenic environmental changes affect ecosystem stability via biodiversity. Science 348, 336–339 (2015). 25883357 10.1126/science.aaa1788

[b41] Castello L. . The vulnerability of Amazon freshwater ecosystems. Conserv. Lett. 6, 217–229 (2013).

[b42] Renó V. F. . Assessment of deforestation in the Lower Amazon floodplain using historical Landsat MSS/TM imagery. Remote Sens. Environ. 115, 3446–3456 (2011).

[b43] Pinaya W. H. D. . Multispecies fisheries in the lower Amazon River and its relationship with the regional and global climate variability. PLoS One 11, e0157050 (2016). 27314951 10.1371/journal.pone.0157050 PMC4912141

[b44] Pires T. H. S. . Ecology and life-history of *Mesonauta festivus*: biological traits of a broad ranged and abundant Neotropical cichlid. Environ. Biol. Fishes 98, 789–799 (2015).

[b45] Neves Dos Santos R., Ferreira E. J. G. & Amadio S. A. Effect of seasonality and trophic group on energy acquisition in Amazonian fish. Ecol. Freshw. Fish 17, 340- 348 (2008).

[b46] Bittencourt M. M. & Amadio S. A. Proposta para identificação rápida dos períodos hidrológicos em áreas de várzea do rio Solimões-Amazonas nas proximidades de Manaus. Acta Amazonica 37, 303–308 (2007).

[b47] Oksanen J. *et al. vegan: Community Ecology Package*. R package version 2.2-0 (2014).

[b48] R Development Core Team. R: A language and environment for statistical computing. R Foundation for Statistical Computing, Vienna, Austria. ISBN 3-900051-07-0 (2014).

[b49] Rodionov S. N. A sequential algorithm for testing climate regime shifts. Geophys. Res. Lett. 31, L09204 (2004).

[b50] Rodionov S. N. Use of prewhitening in climate regime shift detection. Geophys. Res. Lett. 33, L12707 (2006).

[b51] Nicholls K. H. Detection of regime shifts in multi-species communities: the Bay of Quinte phytoplankton example. Methods Ecol. Evol. 2, 416–426 (2011).

[b52] Winemiller K. O. & Rose K. A. Patterns of life-history diversification in North American fishes: implications for population regulation. Can. J. Fish Aquat. Sci. 49, 2196–2218 (1992).

[b53] Winemiller K. O. Patterns of variation in life history among South American fishes in seasonal environments. Oecologia 81, 225–241 (1989). 10.1007/BF00379810 28312542

[b54] Legendre P. & Gallagher E. Ecologically meaningful transformations for ordination of species data. Oecologia 129, 271–280 (2001). 10.1007/s004420100716 28547606

[b55] Andersen T., Carstensen J., Hernández-García E. & Duarte C. M. Ecological thresholds and regime shifts: approaches to identification. Trends Ecol. Evol. 24, 49–57 (2008). 18952317 10.1016/j.tree.2008.07.014

[b56] Zuur A. F., Ieno E. N. & Smith G. M. Analysing Ecological Data (Springer-Verlag, New York, 2007).

[b57] Pinheiro J., Bates D., DebRoy S. & Sarkar D. *R* Core Team, *nlme*: Linear and Nonlinear Mixed Effects Models. R package version 3.1-120 (2015).

[b58] Mazerolle M. J. AICcmodavg: Model selection and multimodel inference based on (Q)AIC(c). R package version 2.0-3 (2015).

