# Supplementary material for: Simultaneous abrupt shifts in hydrology and fish assemblage structure in a floodplain lake in the central Amazon
Source: Sci Rep. 2017 Jan 10;7:40170. doi: 10.1038/srep40170 (PMC5223205; doi:10.1038/srep40170)
Supplement: Supplementary Information [file srep40170-s1.pdf]

SUPPLEMENTARY INFORMATION

Simultaneous abrupt shifts in hydrology and fish assemblage structure in a floodplain lake in central Amazon

Cristhiana P. Röpke[ab1], Sidinéia Amadio[c], Jansen Zuanon[c], Efrem J. G. Ferreira[c], Cláudia Pereira de Deus[c], Tiago H. da S. Pires[a], Kirk O. Winemiller[d]

[a]Programa de Pós-Graduação em Biologia de Água Doce e Pesca Interior, Instituto Nacional de Pesquisas da Amazônia, Manaus, Amazonas, 69067-375, Brasil.

[b]Programa de Pós-Graduação em Ciências Pesqueiras nos Trópicos, Faculdade de Ciências Agrárias, Universidade Federal do Amazonas, Manaus, Amazonas, 69077-000, Brasil.

[c]Coordenação de Biodiversidade, Instituto Nacional de Pesquisas da Amazônia, Manaus, Amazonas, 69067-375, Brasil.

[d]Department of Wildlife and Fisheries Sciences, Texas A&M University, College Station, TX, 77843-2258, USA

[1]To whom correspondence should be addressed: Cristhiana P. Röpke

krikaropke@gmail.com

Av. André Araújo, 2936, Laboratório de Dinâmica de Populações, Instituto Nacional de Pesquisas da Amazônia – INPA, ZIP 69080-971, Manaus, AM, Brasil.

Supplementary Material and Methods

Study area

This study was carried out in the Lago Catalão, an Amazonian floodplain lake located near Manaus at the confluence of the Amazonas and Negro rivers (3°08'– 3°14' S and 59°53'–59°58' W) (Fig. 1a). This region encompasses an annual cyclic water level fluctuation that is driven by the rainfall regime in the west, northwest and southwest of the Amazonian basin, naturally controlled by climatic events. The hydrometric regime result in four hydrometric moments (seasons) of the flood cycle in this region: rising water, typically between January and April; flood, between May and July; receding water, between August and September; and dry, between October and December (46, 59).

Seasonal water level fluctuation implies changes in the connectivity of the lake with both rivers during the flood season and may be totally isolated during the dry season (60). This processes determines the limnological characteristics of the water in the lake which varies pronouncedly intra-annually; during the dry season the decomposition of the aquatic macrophytes stand increases the water conductivity (60) and productivity (61, 62) which results in reduction of the dissolved oxygen, during this period the density of fishes also increase (35). The absence of white water flux from Amazonas River also contribute for decrease the pH during the dry season and increase during the flood when reconnected to Amazonas River (60, 63). The annual connectivity of the lake with the main channel of both Amazonas and Negro rivers increase the probability of fish species to disperse and recolonize, this results in a great abundance of seasonally transient species and assemblages noticeably different between dry and flood seasons (35).

Fish surveys and ecological information. For the present investigation, we analyzed data from surveys from October 1999 until October 2014; this period represents 15 flood pulses cycles with monthly surveys. The dataset has two gaps caused by logistics problems that resulted in incomplete sampling during those intervals: October 2001 to September 2002 and October 2005 to November 2006. Consequently, those two intervals were not included in our analyses. Fish were sampled using sets of 10 gillnets of different mesh sizes, with a monthly fishing effort of 257.26 m^2 x 24 hours. All fishes were removed from gillnets every 6 hours, and euthanized in an ice bath and transported in boxes of ice (details appear in 35). Fish surveys were authorized by IBAMA through license #101932, and procedures followed INPA's ethics committee rules (protocol number 33/2012). In the laboratory, each specimen was identified, measured for standard length (SL, mm), weighed (g), and sexed. Fecundity was estimated by counting all oocytes found in the most developed batch (batch fecundity) using the gravimetric method. Oocyte diameter was measured by stereomicroscope in millimeters (mm) (detailed methods appear in 44). Dietary analysis was based on frequency of occurrence and relative volume of food items present in the stomach (detailed methods appear in 45).

Hydrology – annual flood-pulse attributes. Daily records of the water level in the Negro River since 1950 were obtained from the website of Porto de Manaus Company (http://www.portodemanaus.com.br) and National Agency for Water (http:// http://hidroweb.ana.gov.br). Temporal components of the annual flood pulse were defined based on analysis of water level recorded over 100 years (46). Dry season was defined as the period when the stage level is lower than 20 m; the rising season was when the water level is between 20 m and 26 m with an ascendant vector (increasing water level); flood

season was when the stage level is higher than 26 m; and the receding season was when the stage level is between 26 m and 20 m with a descendant vector (lowering water level) (46). Each annual flood-pulse was defined as beginning during the dry season of a given year and ending during the subsequent dry season. This was an interval from when the ascending water level was at 20 m until it had peaked and declined again to 20 m at the beginning of the next dry season. Seven variables were used to describe hydrological dynamics: amplitude of the flood pulse; annual lowest water level (stage); annual highest water level; number of days of the dry season; number of days of the rising season; number of days of the flood season; and number of days of the receding season (Dataset S1). Principal components analysis (PCA) was used to ordinate years according to hydrological variables. Prior to analysis, data were standardized using *decostand* function in *vegan* library (47); PCA was performed using *stats* library and *prcomp* function (47), in R software (48). The number of axes considered for interpretation and further analysis was delimited by Scree Plot and Broken Stick methods. For detection of changes on hydrological time series, the Sequential T-test Analysis of Regime-Shifts algorithm (STARS) (49, 50) was applied to the ordination data (axis 1 and 2) of the PCA, which modeled 82% of total variation. Time-series data were passed through a white-noise filter using the ordinary least-squares method (49, 50). We set the p value at 0.05, and the window size to identify significant change points was set to 10 years.

Fish assemblage structure. Data used to infer assemblage taxonomic and functional structures and patterns of population abundance were coded according to annual flood pulse cycles as defined for the hydrological analysis (i.e., an annual cycle was from the beginning of the dry season during a given year until the beginning of the next dry season). Each cycle

included monthly surveys covering all seasons of the flood pulse (dry, rising, flood, and receding seasons).

Taxonomic structure - To ensure consistency of species identity in the light of new taxonomic revisions, we retained the taxonomic nomenclature used at the beginning of the project (e.g., not splitting previously identified species into multiple species, which would bias the analysis by increasing taxonomic diversity over time). Rare species with low abundance (< 0.01 individual/survey unit) were omitted because they can bias results in multivariate statistical analysis. In total, 97 common species out of 196 total species were included in the data matrix for analysis. To avoid potential bias from inter-annual differences in sampling effort resulting of the length of each complete flood cycle (e.g., differences in the total number of months to complete the flood pulse cycle, for instance the cycle of 2004-2005 lasted 10 months and 2002-2003 lasted 13 months; the length of each cycle in months can be found in the Dataset S2), abundance data were transformed to catch per unit effort (CPUE) (Dataset S2). Similarities in assemblage composition among annual flood pulses were investigated by principal coordinates analysis (PCoA). Prior to analysis, data were square root transformed to reduce the influence of abundant species (51) and converted into a Bray-Curtis distance matrix using *vegdist* function, in *vegan* library. PCoA analysis was performed using *stats* and *BiodiversityR* libraries using functions *cmdscale* and *add.spec.scores*.

Life history - For all 97 species, we obtained information on life history traits that were considered likely to influence how populations respond to patterns of environmental variation (30, 52, 53). Most information was species-specific; whenever specific information was not available, we used available data for the genus or for the closest related genus, if information was lacking at the genus level (this last case represents 6% of the total

species). We assessed life history trait information from multiple sources, including primary data collected during our long-term research project at Lago Catalão, and short term projects in Rio Uatumã (Negro River Basin) and Lago do Rei (Amazon floodplain), and published scientific papers (Dataset S3; Refs. 44, 45, 64-88). Life history traits were as follows: maximum size - SL for the largest specimen captured in our surveys or the maximum size reported in the literature (when data from our project clearly underestimated the maximum size for the species); size at sexual maturation - SL of the smallest ripe female captured in our surveys or the L_{50} value when this information was available in the literature; fecundity - number of oocytes in the cohort with greatest oocyte diameter within a mature ovary, representing the ripe cohort to be released in the next reproductive event; oocyte diameter – mean diameter based on the cohort of largest oocytes in mature ovaries; degree of parental care - was classified according to scores (ranging from 0 to 6) representing a summation of degrees of investment in individual offspring 53): total absence of parental care (0); special placement of zygotes (1); special placement of zygotes and brief period of parental care by one sex (2); special placement of zygotes and brief period of parental care by both sexes (3); special placement of zygotes and long period of parental care of larvae and juveniles by one sex (4); special placement of zygotes and long period of parental care of larvae and juveniles by both sex (6). We performed PCA using life-history traits data to ordinate species along a life history strategy continuum (52, 53). Prior to analysis, data were natural logarithm transformed and standardized. From the resulting correlations between species traits and PC axis scores, we identified five groups among species ordinated by the fecundity and parental care gradients (PCA 1- 50%), and secondarily by oocyte diameter, maximum size, and size at sexual maturation gradients (PCA 2 - 28%) (supplementary Fig. S3):

- *Equilibrium-small* (ES, 12 species) - small batch fecundity (<3,000); large oocytes diameter (1.5 – 12 mm); well-developed parental care (score values ≥4); maturation size <120 mm SL; and maximum size 97 – 269 mm SL;

- *Equilibrium-large* (EL, 3 species) - small batch fecundity (<3,000); large oocytes diameter (1.5 – 12 mm); well-developed parental care (score values ≥4); maturation size >170 mm SL; and maximum size >400 mm SL;

- *Intermediate-small* (IS, 18 species) - batch fecundity 1,000 – 9,000; large oocytes diameter (1.4 – 2 mm); parental care less developed relative to equilibrium strategists but more so than in periodic strategists;

- *Periodic-large* (PL, 29 species) - maturation size >164 mm SL; maximum size >253 mm SL; average batch fecundity about 90,000 oocytes; small oocyte diameter (0.7 – 1.6 mm); no parental care; *Rhamphicthys marmoratus* was classified as PL due to large size and absence of parental care but its batch fecundity was lower for this groups (1,000 oocytes).

- *Periodic-small* (PS, 35 species) - maturation size 63 – 148 mm SL; maximum size 137 – 410 mm SL; batch fecundity 6,762 – 74,227; small oocyte diameter (0.5 – 1.3 mm); no parental care.

Opportunistic strategists (i.e., small size, low fecundity, small oocytes, little or no parental care) were nearly absent our samples because they were too small (<60 mm SL) to be captured by the standardized sampling method (gillnets).

Trophic level - Classification of species into vertical trophic positions was based on stomach contents data collected during three years of our long-term research project (years 1, 4 and 9) and supplemented with information from the published literature (Dataset 3; Refs. 44, 45, 64-88). Each species was then assigned one of the four trophic levels:

- *Consumer Trophic Level 1*- herbivorous and detritivorous species (n= 26) that predominantly ingest plant material (seeds, fruits or leaves), filamentous algae and fine particulate organic matter that in the Amazon originates mostly from periphyton (87, 88);
- *Consumer Trophic Level 1.5*- omnivorous species (n= 21) that ingest combinations of animal, plant, and detritus;
- *Consumer Trophic Level 2*- invertivorous and planktivorous species (n= 26) that predominantly ingest insects (adults or immature forms of aquatic and terrestrial insects), microcrustaceans from the benthos or water column, spiders; shrimps, and/or molluscs;
- *Consumer Trophic Level 3*- piscivorous species (n= 24) that ingest adult, juvenile or larval fish, either whole or pieces such as scales and fins.

Supplementary Figures

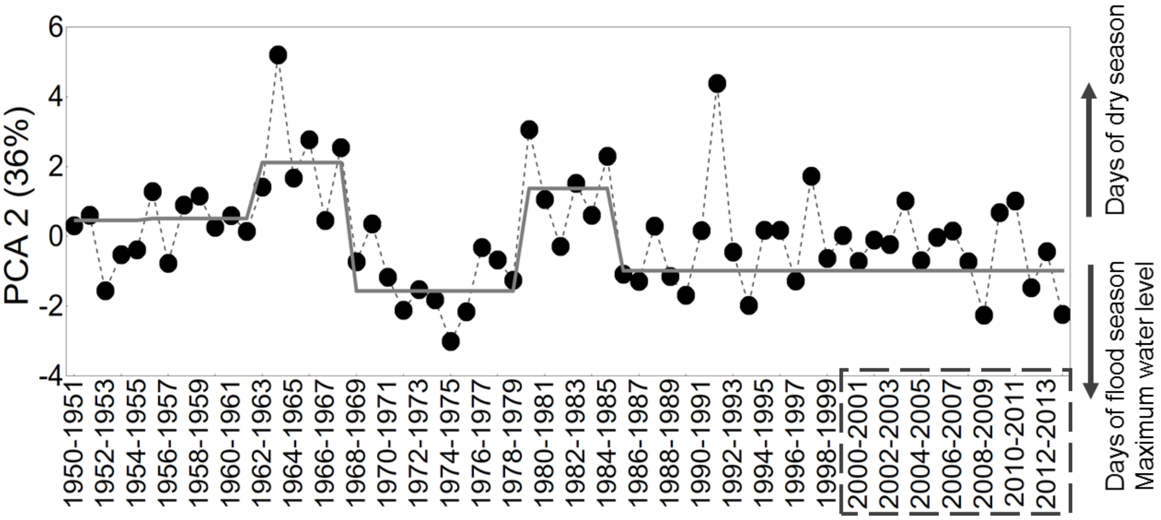

Fig. S1. Temporal dynamics of hydrology, PCA 2 ordination based on 7 variables describing each flood pulse cycle (supplementary Material and Methods, Dataset S1). Mean scores value is represented by the continuous grey line. Mean change shows the moments of significant hydrological changes ($P<0.05$) which occurred four times (1957, 1962, 1969, 1979, 1986).

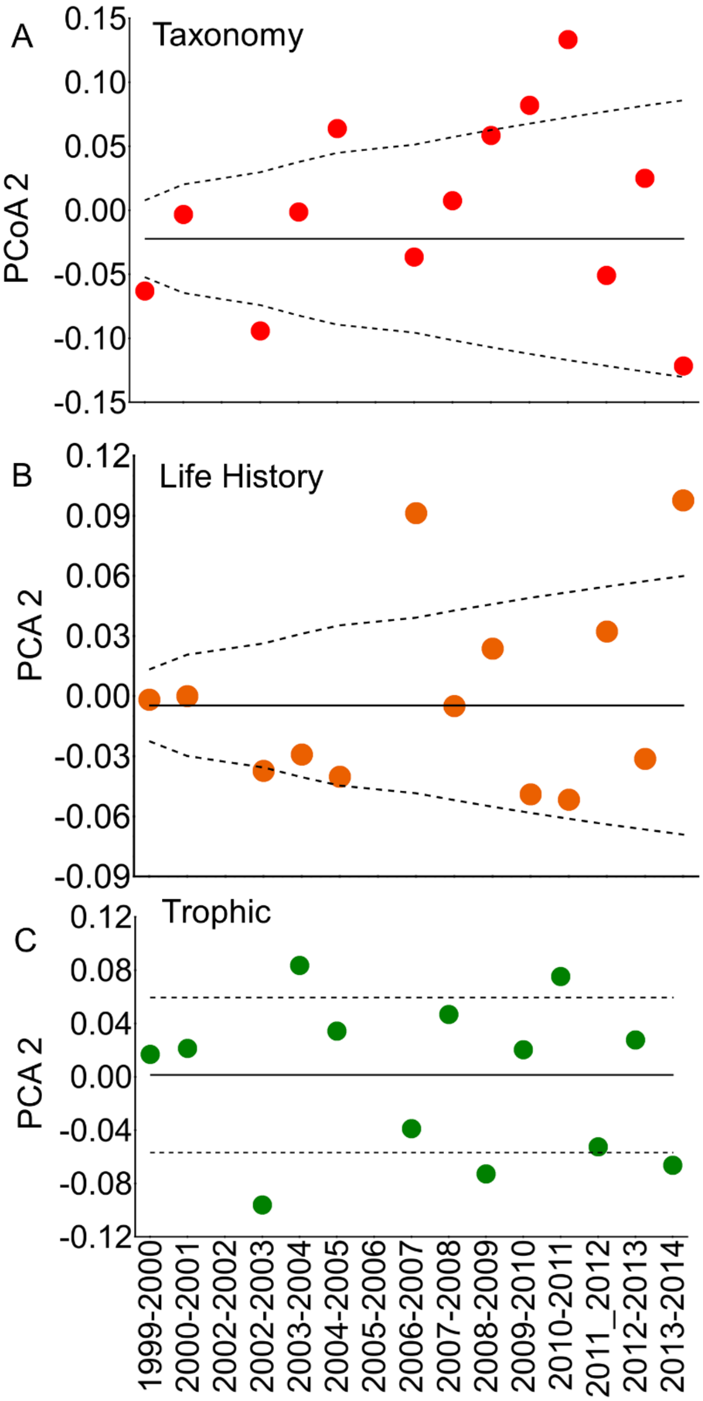

Fig. S2. Temporal dynamics of assemblage structures as defined by scores on the second axis from PCoA or PCA. Solid lines represent best-fit regression models describing time series (Table S2); dashed lines represent standard errors around mean estimates.

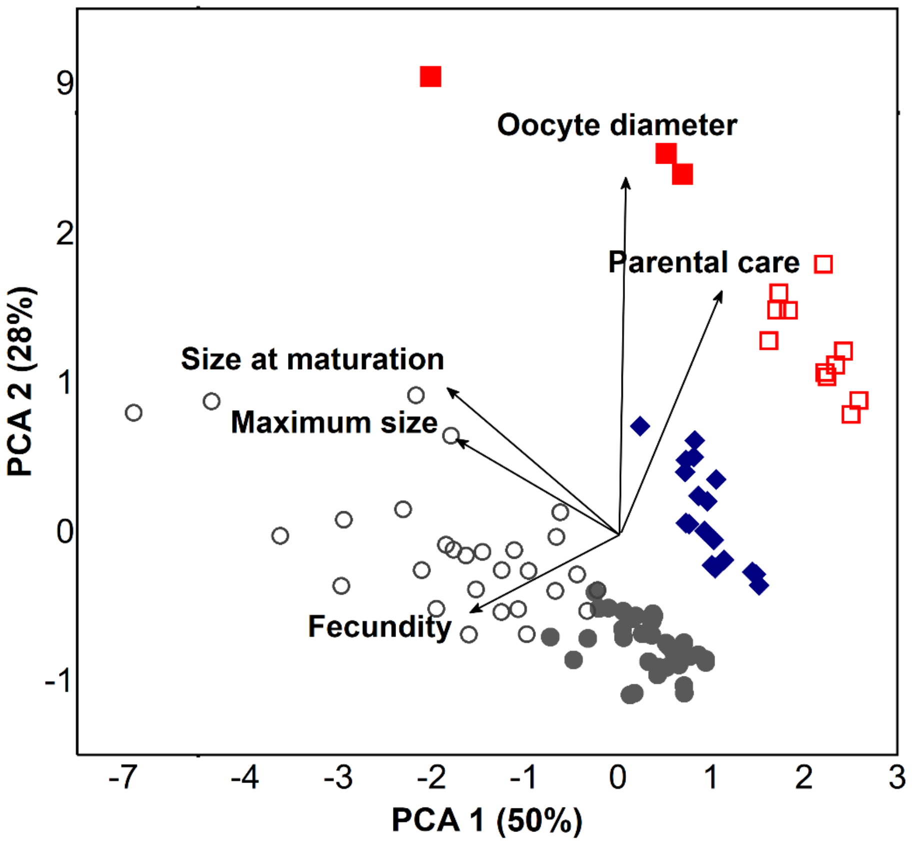

Fig. S3. Principal components analysis with species ordination according to values for life history traits. Species life history classification: EL- equilibrium-large (solid squares); ES- equilibrium-small (open squares); IS- intermediate-small (diamonds); PL- periodic-large (dots); PS- periodic-small (circles) (Dataset S3).

Supplementary Tables

Table S1: Significant change points indicated by STARS (Sequential T-test Analysis of Regime-Shifts) and variance explained by multivariate statistics for years in term of hydrological and biotic variables. Cut-off length (window size) and significance level parameters was 10 years and 0.05 for hydrology, and 7 years and 0.01 for biotic variables.

Variable	Axis	Axis explanation (%)	STARS	
			Change Point	*P*
Hydrology	PCA 1	46	1971	<0.001
			1987	<0.01
			2005	<0.05
	PCA 2	36	1956	<0.05
			1962	<0.05
			1969	<0.001
			1979	<0.01
			1986	<0.01
Taxonomic structure	PCoA 1	34	2007	<0.001
	PCoA 2	16	2007	<0.05
Life history structure	PCA 1	59	2007	<0.001
	PCA 2	23	2003	<0.01
Trophic structure	PCA 1	63	2007	<0.001
	PCA 2	30	…	…
CPUE	Equilibrium-small		2007	<0.001
	Equilibrium-large		2007	0.01
	Intermediate-small		2007	<0.001
	Periodic-large		2007	<0.01
	Periodic-small		2007	<0.05
	Consumer trophic level 1		…	…
	Consumer trophic level 1.5		2007	<0.001
	Consumer trophic level 2		2007	<0.01
	Consumer trophic level 3		2007	<0.001

Table S2: Results for non-linear least squares regression fitted models; the best models were tested controlling the heteroscedasticity of standard error (+VAR) and temporal autocorrelation (+AR) components in the model. AICc - Akaike information criterion with a correction for finite sample sizes; LL - Log-likelihood; RSE - Residual standard error. The best-fit models are in bold.

y	x	Model	Number of parameters	AICc	LL	RSE
PCoA 1 Taxonomic assemblage structure	Years	Null	2	-16.931	11.0657	0.107
		Linear	3	-31.094	19.88	0.056
		Segmented Linear- Linear	5	-24.373	21.472	0.055
		**Segmented Step- Mean**	**4**	**-36.954**	**22.81**	**0.045**
		Segmented Step- Mean + VAR	5	-35.25	21.968	0.024
		Segmented Step- Mean + AR	5	-35.864	24.432	0.049
		Segmented Linear, Stable Mean	4	-24.672	16.669	0.072
		Segmented Stable- Mean, Linear	4	-22.973	15.82	0.077
		Sigmoidal	5	-14.66	16.616	0.081
PCoA 2 Taxonomic assemblage structure	Years	Null	2	-26.924	16.064	0.073
		**Null + VAR**	**3**	**-27.584**	**16.392**	**0.029**
		Null + AR	3	-23.501	16.084	0.073
		Linear	3	-23.741	16.204	0.075
		Segmented Linear- Linear	5	DNC	DNC	DNC
		Segmented Step- Mean	4	-25.937	17.301	0.069
		Segmented Linear, Stable Mean	4	-24.271	16.468	0.074
		Segmented Stable- Mean, Linear	4	-23.531	16.099	0.076
		Sigmoidal	5	-4.24	11.407	0.102
PCA 1 Life history assemblage structure	Years	Null	2	-24.82	15.01	0.079
		Linear	3	17.216	17.216	0.069

		Segmented Linear- Linear	5	-23.106	20.838	0.058
		Segmented Step- Mean	4	-30.097	19.382	0.059
		**Segmented Step- Mean + VAR**	**5**	**-32.693**	**20.68**	**0.022**
		Segmented Step- Mean + AR	5	-28.397	20.698	0.059
		Segmented Linear, Stable Mean	4	-23.07	15.868	0.077
		Segmented Stable- Mean, Linear	4	-24.833	16.749	0.072
		Sigmoidal	5	-12.232	15.402	0.088
		Null	2	-37.093	21.146	0.049
		**Null + VAR**	**3**	**-41.012**	**23.106**	**0.017**
		Null + AR	3	-34.487	21.576	0.049
		Linear	3	-34.427	21.55	0.052
PCA 2 Life history assemblage structure	Years	Segmented Linear- Linear	5	DNC	DNC	DNC
		Segmented Step- Mean	4	-35.429	22.05	0.049
		Segmented Linear, Stable Mean	4	-33.871	21.27	0.053
		Segmented Stable- Mean, Linear	4	-34.31	21.49	0.052
		Sigmoidal	5	-11.382	14.98	0.094
		Null	2	-23.119	14.159	0.084
		Linear	3	-20.907	14.787	0.083
		Segmented Linear- Linear	5	-20.065	19.318	0.065
		Segmented Step- Mean	4	-26.339	17.501	0.068
PCA 1 Trophic assemblage structure	Years	**Segmented Step- Mean + VAR**	**5**	**-31.354**	**20.01**	0.023
		Segmented Step- Mean + AR	5	-22.163	17.581	0.068
		Segmented Linear, Stable Mean	4	-23.336	15.966	0.077
		Segmented Stable- Mean, Linear	4	-19.668	14.167	0.088
		Sigmoidal	5	-9.978	14.274	0.096
		Null	2	-32.743	18.971	0.058
PCA 2 Trophic assemblage structure	Years	Null + VAR	**3**	-31.508	18.354	0.025
		**Null + AR**	3	**-33.284**	**20.975**	0.058

		Linear	3	-29.529	19.098	0.065
		Segmented Linear- Linear	5	DNC	DNC	DNC
		Segmented Step- Mean	4	-29.743	19.204	0.06
		Segmented Linear, Stable Mean	4	-29.502	19.084	0.06
		Segmented Stable- Mean, Linear	4	-29.629	19.148	0.06
		Sigmoidal	5	-11.047	14.809	0.093
CPUE – Equilibrium-small	Years	Null	2	-56.472	30.84	0.023
		Linear	3	-68.153	38.41	0.013
		Segmented Linear- Linear	5	-60.717	39.64	0.013
		**Segmented Step- Mean**	**4**	**-71.109**	**39.89**	**0.012**
		Segmented Step- Mean + VAR	5	-67.332	37.999	0.005
		Segmented Step- Mean + AR	5	-67.112	40.076	0.012
		Segmented Linear, Stable Mean	4	-60.205	34.44	0.018
		Segmented Stable- Mean, Linear	4	-63.243	35.96	0.016
		Sigmoidal	5	-61.301	39.65	0.013
CPUE – Equilibrium-large	Years	Null	2	-78.079	41.63	0.102
		Linear	3	-79.722	44.194	0.008
		Segmented Linear- Linear	5	-70.254	44.128	0.009
		**Segmented Step- Mean**	**4**	**-86.709**	**47.688**	**0.006**
		Segmented Step- Mean+ VAR	5	-72.443	40.554	0.004
		Segmented Step- Mean + AR	5	-81.725	47.362	0.006
		Segmented Linear, Stable Mean	4	76.313	42.49	0.01
		Segmented Stable- Mean, Linear	4	-78.773	43.72	0.009
		Sigmoidal	5	-62.42	40.495	0.012
CPUE – Intermediate-small	Years	Null	2	-23.794	14.479	0.082
		Linear	3	-29.091	18.879	0.061
		Segmented Linear- Linear	5	DNC	DNC	DNC
		**Segmented Step- Mean**	**4**	**-34.516**	**21.591**	**0.049**

		Segmented Step- Mean + VAR	5	-33.094	20.45	0.022
		Segmented Step- Mean + AR	5	-33.245	22.584	0.051
		Segmented Linear, Stable Mean	4	-26.135	15.899	0.068
		Segmented Stable- Mean, Linear	4	-25.671	17.401	0.07
		Sigmoidal	5	-13.227	17.169	0.085
CPUE – Periodic-large	Years	Null	2	-31.819	18.51	0.06
		Linear	3	-43.772	26.22	0.035
		Segmented Linear- Linear	5	DNC	DNC	DNC
		Segmented Step- Mean	4	-47.4	28.03	0.034
		**Segmented Linear, Stable Mean**	**4**	**-52.881**	**30.77**	**0.024**
		Segmented Linear, Stable Mean + VAR	5	-42.685	27.842	0.213
		Segmented Linear, Stable Mean + AR	5	-37.114	25.057	0.038
		Segmented Stable- Mean, Linear	4	-40.601	27.92	0.039
		Sigmoidal	5	-37.269	24.63	0.033
CPUE – Periodic-small	Years	Null	2	0.254	2.47	0.208
		Linear	3	-0.929	4.8	0.181
		Segmented Linear- Linear	5	DNC	DNC	DNC
		Segmented Step- Mean	4	-0.391	4.53	0.185
		Segmented Linear, Step Mean	4	2.175	3.25	0.204
		Segmented Step- Mean, Linear	4	-0.958	4.81	0.181
		Segmented Stable- Mean, Linear + VAR	5	-0.294	4.48	0.078
		**Segmented Stable- Mean, Linear + AR**	**5**	**-1.012**	**7.006**	**0.185**
		Sigmoidal	5	DNC	DNC	DNC
CPUE – Consumer Trophic Level 1	Years	Null	2	-20.14	12.67	0.095
		Linear	3	-18.38	13.53	0.092
		Segmented Linear- Linear	5	-16.54	14.77	0.088

		Segmented Step- Mean	4	-18.02	13.35	0.094
		Segmented Linear, Step Mean	**5**	-16.73	12.7	0.099
		Segmented Step- Mean, Linear	5	-20.37	14.52	0.086
		**Segmented Stable- Mean, Linear + VAR**	4	**-21.585**	15.126	0.034
		Segmented Stable- Mean, Linear + AR	4	-16.514	14.757	0.096
		Sigmoidal	5	-6.64	12.61	0.11
		Null	2	-7.31	6.26	0.155
		Linear	3	-9.7	9.18	0.129
		Segmented Linear- Linear	5	-5.37	9.18	0.136
		**Segmented Step- Mean**	4	**-11.5**	**10.09**	**0.121**
CPUE – Consumer Trophic Level 1.5	Years	Segmented Step- Mean+ VAR	**5**	-8.727	8.697	0.056
		Segmented Step- Mean + AR	5	-11.325	12.162	0.122
		Segmented Linear, Stable Mean	4	-7.17	7.92	0.143
		Segmented Stable- Mean, Linear	4	-8.08	8.37	0.138
		Sigmoidal	5	0.86	8.85	0.143
		Null	2	-26.05	15.63	0.075
		Linear	3	-33.25	20.96	0.052
		Segmented Linear- Linear	5	-30.25	21.63	0.052
		**Segmented Step- Mean**	4	**-40.94**	**24.8**	0.039
CPUE – Consumer Trophic Level 2	Years	Segmented Step- Mean+ VAR	**5**	-39.851	26.425	0.065
		Segmented Step- Mean + AR	5	37.671	25.335	0.039
		Segmented Linear, Stable Mean	4	-31.91	20.29	0.055
		Segmented Stable- Mean, Linear	4	-27.94	18.31	0.064
		Sigmoidal	5	-19.94	19.26	0.066
CPUE – Consumer Trophic Level 3	Years	Null	2	-27	16.1	0.072
		Linear	3	-38.57	23.62	0.042

Segmented Linear- Linear	5	-33.63	26.1	0.039
Segmented Step- Mean	4	-42.83	25.75	0.036
**Segmented Linear, Stable Mean**	**4**	**-42.91**	**25.79**	**0.036**
Segmented Linear, Stable Mean + VAR	5	-38.721	25.86	0.036
Segmented Linear, Stable Mean + AR	5	-40.177	26.588	0.037
Segmented Stable- Mean, Linear	4	-36.94	22.8	0.045
Sigmoidal	5	-24.72	21.64	0.055

Table S3: AICc values for models simulating all possible values for 2005-2006 on a 0.001 increment within the range of the coordinates in the axis 1 for taxonomic structure of the Lago Catalão fish assemblage. 98% of the best-fit model was segmented step-mean.

Possible values	Null	Linear	Segmented linear-linear	Segmented step mean	Segmented linear-stable mean	Segmented stable mean-linear	Sigmoidal
-0.172	-17.262	-31.347	-28.549	**-40.441**	-22.604	-37.822	-17.704
-0.171	-17.289	-31.420	-28.636	**-40.489**	-22.599	-37.870	-17.828
-0.170	-17.316	-31.493	-28.724	**-40.536**	-22.594	-37.918	-17.951
-0.169	-17.342	-31.566	-28.811	**-40.583**	-22.589	-37.965	-18.075
-0.168	-17.369	-31.639	-28.899	**-40.629**	-22.584	-38.012	-18.199
-0.167	-17.395	-31.711	-28.986	**-40.674**	-22.579	-38.058	-18.324
0.166	-17.421	-31.783	-29.073	**-40.718**	-22.573	-38.103	-18.448
-0.165	-17.448	-31.855	-29.160	**-40.762**	-22.567	-38.148	18.573
-0.164	-17.474	-31.927	-29.247	**-40.804**	22.561	-38.192	-18.699
-0.163	-17.500	-31.999	-29.335	**-40.846**	-22.555	-38.235	-18.824
-0.162	-17.525	-32.070	-29.422	**-40.887**	-22.549	-38.278	-18.950
-0.161	-17.551	-32.141	-29.509	**-40.927**	-22.543	-38.320	-19.076
-0.160	-17.577	-32.212	-29.596	**-40.966**	-22.536	-38.361	-19.202
-0.159	-17.602	-32.283	-29.682	**-41.005**	-22.530	-38.402	-19.328
-0.158	-17.628	-32.354	-29.769	**-41.042**	-22.523	-38.442	-19.455
-0.157	-17.653	-32.424	-29.856	**-41.079**	-22.516	-38.481	-19.582
-0.156	-17.678	-32.494	-29.942	**-41.115**	-22.508	-38.519	-19.709
-0.155	-17.703	-32.564	-30.029	**-41.150**	-22.501	-38.557	-19.836
-0.154	-17.728	-32.634	-30.115	**-41.184**	-22.494	-38.594	-19.963
-0.153	-17.753	-32.703	-30.202	**-41.217**	-22.486	-38.630	-20.091
-0.152	-17.777	-32.772	-30.288	**-41.249**	-22.478	-38.666	-20.219
-0.151	-17.802	-32.841	-30.374	**-41.280**	-22.470	-38.700	-20.347
-0.150	-17.826	-32.910	-30.460	**-41.311**	-22.462	-38.735	-20.475
-0.149	-17.851	-32.978	-30.546	**-41.340**	-22.454	-38.768	-20.603
-0.148	-17.875	-33.046	-30.632	**-41.368**	-22.445	-38.800	-20.732
-0.147	-17.899	-33.114	-30.717	**-41.396**	-22.437	-38.832	-20.860
-0.146	-17.923	-33.182	-30.803	**-41.423**	-22.428	-38.863	-20.989
-0.145	-17.947	-33.249	-30.888	**-41.448**	-22.419	-38.893	-21.118
-0.144	-17.971	-33.316	-30.974	**-41.473**	-22.410	-38.923	-21.247
-0.143	-17.994	-33.383	-31.059	**-41.497**	-22.400	-38.951	-21.376
-0.142	-18.018	-33.449	-31.143	**-41.519**	-22.391	-38.979	-21.505
-0.141	-18.041	-33.515	-31.228	**-41.541**	-22.381	-39.006	-21.635
-0.140	-18.064	-33.581	-31.313	**-41.562**	-22.372	-39.033	-21.764
-0.139	-18.087	-33.647	-31.397	**-41.582**	-22.362	-39.058	-21.893
-0.138	-18.110	-33.712	-31.481	**-41.601**	-22.352	-39.083	-22.023

-0.137	-18.133	-33.776	-31.565	**-41.619**	-22.342	-39.106	-22.152
-0.136	-18.156	-33.841	-31.649	**-41.635**	-22.331	-39.129	-22.282
-0.135	-18.178	-33.905	-31.733	**-41.651**	-22.321	-39.152	-22.412
-0.134	-18.201	-33.969	-31.816	**-41.666**	-22.310	-39.173	-22.541
-0.133	-18.223	-34.032	-31.899	**-41.680**	-22.299	-39.193	-22.671
-0.132	-18.245	-34.095	-31.982	**-41.693**	-22.288	-39.213	-22.800
-0.131	-18.267	-34.158	-32.065	**-41.705**	-22.277	-39.232	-22.930
-0.130	-18.289	-34.220	-32.147	**-41.716**	-22.266	-39.250	-23.059
-0.129	-18.311	-34.282	-32.230	**-41.725**	-22.254	-39.267	-23.189
-0.128	-18.333	-34.343	-32.312	**-41.734**	-22.243	-39.283	-23.318
-0.127	-18.354	-34.405	-32.393	**-41.742**	-22.231	-39.299	-23.447
-0.126	-18.375	-34.465	-32.475	**-41.749**	-22.219	-39.314	-23.576
-0.125	-18.397	-34.526	-32.556	**-41.755**	-22.207	-39.327	-23.705
-0.124	-18.418	-34.585	-32.637	**-41.759**	-22.195	-39.340	-23.834
-0.123	-18.439	-34.645	-32.717	**-41.763**	-22.183	-39.352	-23.963
-0.122	-18.459	-34.704	-32.798	**-41.766**	-22.170	-39.363	-24.091
-0.121	-18.480	-34.762	-32.878	**-41.767**	-22.157	-39.374	-24.220
-0.120	-18.501	-34.821	-32.957	**-41.768**	-22.145	-39.383	-24.348
-0.119	-18.521	-34.878	-33.037	**-41.768**	-22.132	-39.392	-24.476
-0.118	-18.541	-34.936	-33.116	**-41.766**	-22.119	-39.399	-24.603
-0.117	-18.561	-34.992	-33.195	**-41.764**	-22.105	-39.406	-24.731
-0.116	-18.581	-35.049	-33.273	**-41.760**	-22.092	-39.412	-24.858
-0.115	-18.601	-35.104	-33.351	**-41.756**	-22.078	-39.417	-24.984
-0.114	-18.621	-35.160	-33.428	**-41.750**	-22.065	-39.421	-25.111
-0.113	-18.640	-35.215	-33.506	**-41.744**	-22.051	-39.424	-25.237
-0.112	-18.660	-35.269	-33.583	**-41.736**	-22.037	-39.427	-25.362
-0.111	-18.679	-35.323	-33.659	**-41.728**	-22.023	-39.428	-25.488
-0.110	-18.698	-35.376	-33.735	**-41.718**	-22.008	-39.429	-25.612
-0.109	-18.717	-35.429	-33.811	**-41.708**	-21.994	-39.429	-25.737
-0.108	-18.736	-35.482	-33.886	**-41.696**	-21.979	-39.428	-25.861
-0.107	-18.754	-35.533	-33.961	**-41.683**	-21.965	-39.426	-25.984
-0.106	-18.773	-35.585	-34.035	**-41.670**	-21.950	-39.423	-26.107
-0.105	-18.791	-35.635	-34.109	**-41.655**	-21.935	-39.419	-26.229
-0.104	-18.809	-35.686	-34.183	**-41.640**	-21.920	-39.415	-26.351
-0.103	-18.827	-35.735	-34.256	**-41.623**	-21.904	-39.409	-26.472
-0.102	-18.845	-35.784	-34.328	**-41.605**	-21.889	-39.403	-26.592
-0.101	-18.863	-35.833	-34.400	**-41.587**	-21.873	-39.396	-26.712
-0.100	-18.881	-35.881	-34.472	**-41.567**	-21.858	-39.388	-26.831
-0.099	-18.898	-35.928	-34.543	**-41.547**	-21.842	-39.379	-26.949
-0.098	-18.915	-35.975	-34.614	**-41.525**	-21.826	-39.369	-27.067
-0.097	-18.932	-36.021	-34.684	**-41.503**	-21.810	-39.358	-27.184
-0.096	-18.949	-36.067	-34.753	**-41.479**	-21.794	-39.346	-27.299
-0.095	-18.966	-36.112	-34.822	**-41.455**	-21.777	-39.334	-27.415

-0.094	-18.983	-36.156	-34.891	**-41.429**	-21.761	-39.321	-27.529
-0.093	-18.999	-36.200	-34.959	**-41.403**	-21.744	-39.307	-27.642
-0.092	-19.016	-36.243	-35.026	**-41.376**	-21.727	-39.291	-27.754
-0.091	-19.032	-36.285	-35.093	**-41.347**	-21.710	-39.276	-27.866
-0.090	-19.048	-36.327	-35.159	**-41.318**	-21.693	-39.259	-27.976
-0.089	-19.064	-36.368	-35.225	**-41.288**	-21.676	-39.241	-28.086
-0.088	-19.079	-36.409	-35.290	**-41.257**	-21.659	-39.223	-28.194
-0.087	-19.095	-36.449	-35.354	**-41.225**	-21.641	-39.204	-28.301
-0.086	-19.110	-36.488	-35.418	**-41.192**	-21.623	-39.184	-28.407
-0.085	-19.126	-36.527	-35.481	**-41.158**	-21.606	-39.163	-28.512
-0.084	-19.141	-36.565	-35.543	**-41.124**	-21.588	-39.141	-28.615
-0.083	-19.156	-36.602	-35.605	**-41.088**	-21.570	-39.118	-28.717
-0.082	-19.170	-36.638	-35.666	**-41.052**	-21.552	-39.095	-28.818
-0.081	-19.185	-36.674	-35.727	**-41.015**	-21.533	-39.071	-28.918
-0.080	-19.199	-36.709	-35.786	**-40.976**	-21.515	-39.046	-29.016
-0.079	-19.213	-36.744	-35.845	**-40.937**	-21.496	-39.020	-29.113
-0.078	-19.227	-36.777	-35.904	**-40.897**	-21.478	-38.993	-29.209
-0.077	-19.241	-36.811	-35.962	**-40.857**	-21.459	-38.966	-29.302
-0.076	-19.255	-36.843	-36.018	**-40.815**	-21.440	-38.938	-29.395
-0.075	-19.269	-36.874	-36.075	**-40.773**	-21.421	-38.909	-29.486
-0.074	-19.282	-36.905	-36.130	**-40.729**	-21.402	-38.879	-29.575
-0.073	-19.295	-36.935	-36.185	**-40.685**	-21.383	-38.848	-29.663
-0.072	-19.308	-36.965	-36.239	**-40.641**	-21.363	-38.817	-29.749
-0.071	-19.321	-36.994	-36.292	**-40.595**	-21.344	-38.785	-29.833
-0.070	-19.334	-37.021	-36.344	**-40.548**	-21.324	-38.752	-29.915
-0.069	-19.346	-37.049	-36.396	**-40.501**	-21.304	-38.718	-29.996
-0.068	-19.359	-37.075	-36.447	**-40.453**	-21.284	-38.684	-30.075
-0.067	-19.371	-37.101	-36.497	**-40.404**	-21.264	-38.648	-30.152
-0.066	-19.383	-37.126	-36.546	**-40.355**	-21.264	-38.613	-30.227
-0.065	-19.395	-37.150	-36.594	**-40.305**	-21.290	-38.576	-30.300
-0.064	-19.407	-37.173	-36.642	**-40.254**	-21.316	-38.539	-30.371
-0.063	-19.418	-37.196	-36.688	**-40.202**	-21.342	-38.501	-30.440
-0.062	-19.430	-37.217	-36.734	**-40.149**	-21.368	-38.462	-30.508
-0.061	-19.441	-37.238	-36.779	**-40.096**	-21.393	-38.422	-30.573
-0.060	-19.452	-37.259	-36.823	**-40.042**	-21.418	-38.382	-30.636
-0.059	-19.463	-37.278	-36.866	**-39.988**	-21.443	-38.341	-30.697
-0.058	-19.473	-37.297	-36.908	**-39.932**	-21.468	-38.299	-30.755
-0.057	-19.484	-37.315	-36.950	**-39.877**	-21.492	-38.257	-30.812
-0.056	-19.494	-37.332	-36.990	**-39.820**	-21.516	-38.214	-30.866
-0.055	-19.504	-37.348	-37.030	**-39.763**	-21.540	-38.171	-30.918
-0.054	-19.514	-37.363	-37.068	**-39.705**	-21.564	-38.126	-30.967
-0.053	-19.524	-37.378	-37.106	**-39.646**	-21.587	-38.081	-31.015
-0.052	-19.533	-37.392	-37.143	**-39.587**	-21.611	-38.036	-31.060

-0.051	-19.543	-37.405	-37.178	**-39.527**	-21.634	-37.989	-31.102
-0.050	-19.552	-37.417	-37.213	**-39.467**	-21.657	-37.943	-31.143
-0.049	-19.561	-37.428	-37.247	**-39.406**	-21.679	-37.895	-31.180
-0.048	-19.570	-37.439	-37.280	**-39.344**	-21.702	-37.847	-31.216
-0.047	-19.579	-37.449	-37.312	**-39.282**	-21.724	-37.798	-31.248
-0.046	-19.587	-37.458	-37.343	**-39.219**	-21.746	-37.749	-31.279
-0.045	-19.595	-37.466	-37.373	**-39.156**	-21.768	-37.699	-31.307
-0.044	-19.604	-37.473	-37.402	**-39.092**	-21.789	-37.648	-31.332
-0.043	-19.612	-37.479	-37.430	**-39.027**	-21.810	-37.597	-31.355
-0.042	-19.619	-37.485	-37.456	**-38.962**	-21.831	-37.545	-31.375
-0.041	-19.627	-37.490	-37.482	**-38.897**	-21.852	-37.493	-31.392
-0.040	-19.634	-37.494	-37.507	**-38.830**	-21.873	-37.440	-31.407
-0.039	-19.642	-37.497	-37.531	**-38.764**	-21.893	-37.386	-31.420
-0.038	-19.649	-37.499	-37.554	**-38.697**	-21.913	-37.332	-31.430
-0.037	-19.656	-37.500	-37.576	**-38.629**	-21.933	-37.278	-31.437
-0.036	-19.662	-37.501	-37.596	**-38.561**	-21.952	-37.223	-31.441
-0.035	-19.669	-37.501	-37.616	**-38.492**	-21.972	-37.167	-31.443
-0.034	-19.675	-37.500	-37.635	**-38.423**	-21.991	-37.111	-31.442
-0.033	-19.681	-37.498	-37.652	**-38.354**	-22.010	-37.054	-31.439
-0.032	-19.687	-37.495	-37.669	**-38.284**	-22.028	-36.997	-31.433
-0.031	-19.693	-37.492	-37.684	**-38.213**	-22.046	-36.940	-31.424
-0.030	-19.699	-37.487	-37.698	**-38.143**	-22.065	-36.881	-31.413
-0.029	-19.704	-37.482	-37.712	**-38.071**	-22.082	-36.823	-31.399
-0.028	-19.709	-37.476	-37.724	**-38.000**	-22.100	-36.764	-31.382
-0.027	-19.714	-37.469	-37.735	**-37.927**	-22.117	-36.704	-31.363
-0.026	-19.719	-37.461	-37.745	**-37.855**	-22.134	-36.644	-31.341
-0.025	-19.724	-37.453	-37.754	**-37.782**	-22.151	-36.583	-31.316
-0.024	-19.728	-37.443	**-37.762**	-37.709	-22.168	-36.522	-31.289
-0.023	-19.733	-37.433	**-37.769**	-37.635	-22.184	-36.461	-31.259
-0.022	-19.737	-37.422	**-37.774**	-37.561	-22.200	-36.399	-31.227
-0.021	-19.741	-37.410	**-37.779**	-37.524	-22.216	-36.337	-31.192
-0.020	-19.745	-37.398	**-37.783**	-37.601	-22.232	-36.274	-31.155
-0.019	-19.748	-37.384	**-37.785**	-37.677	-22.247	-36.211	-31.115
-0.018	-19.752	-37.370	**-37.786**	-37.753	-22.262	-36.147	-31.072
-0.017	-19.755	-37.355	-37.787	**-37.828**	-22.277	-36.083	-31.027
-0.016	-19.758	-37.339	-37.786	**-37.903**	-22.291	-36.019	-30.980
-0.015	-19.761	-37.322	-37.784	**-37.978**	-22.305	-35.954	-30.930
-0.014	-19.763	-37.305	-37.781	**-38.052**	-22.319	-35.889	-30.878
-0.013	-19.766	-37.287	-37.777	**-38.126**	-22.333	-35.823	-30.823
-0.012	-19.768	-37.268	-37.771	**-38.199**	-22.347	-35.757	-30.766
-0.011	-19.770	-37.248	-37.765	**-38.272**	-22.360	-35.691	-30.706
-0.010	-19.772	-37.227	-37.758	**-38.344**	-22.373	-35.625	-30.645
-0.009	-19.774	-37.206	-37.749	**-38.416**	-22.385	-35.558	-30.581

-0.008	-19.775	-37.183	-37.740	**-38.488**	-22.398	-35.490	-30.514
-0.007	-19.776	-37.160	-37.729	**-38.559**	-22.410	-35.423	-30.446
-0.006	-19.778	-37.137	-37.717	**-38.629**	-22.422	-35.355	-30.375
-0.005	-19.779	-37.112	-37.705	**-38.699**	-22.433	-35.286	-30.302
-0.004	-19.779	-37.087	-37.691	**-38.768**	-22.445	-35.218	-30.227
-0.003	-19.780	-37.061	-37.676	**-38.837**	-22.456	-35.149	-30.150
-0.002	-19.780	-37.034	-37.660	**-38.905**	-22.466	-35.079	-30.071
-0.001	-19.780	-37.006	-37.643	**-38.973**	-22.477	-35.010	-29.990
0.000	-19.780	-36.978	-37.625	**-39.040**	-22.487	-34.940	-29.907
0.001	-19.780	-36.949	-37.605	**-39.106**	-22.497	-34.870	-29.821
0.002	-19.780	-36.919	-37.585	**-39.172**	-22.507	-34.799	-29.734
0.003	-19.779	-36.889	-37.564	**-39.237**	-22.516	-34.729	-29.645
0.004	-19.779	-36.857	-37.542	**-39.302**	-22.525	-34.658	-29.555
0.005	-19.778	-36.825	-37.518	**-39.366**	-22.534	-34.587	-29.462
0.006	-19.777	-36.793	-37.494	**-39.430**	-22.543	-34.515	-29.368
0.007	-19.775	-36.759	-37.468	**-39.492**	-22.551	-34.443	-29.272
0.008	-19.774	-36.725	-37.442	**-39.554**	-22.559	-34.371	-29.174
0.009	-19.772	-36.690	-37.414	**-39.616**	-22.567	-34.299	-29.074
0.010	-19.770	-36.655	-37.386	**-39.677**	-22.574	-34.227	-28.973
0.011	-19.768	-36.618	-37.357	**-39.737**	-22.581	-34.154	-28.870
0.012	-19.766	-36.581	-37.326	**-39.796**	-22.588	-34.081	-28.766
0.013	-19.764	-36.544	-37.295	**-39.855**	-22.595	-34.008	-28.660
0.014	-19.761	-36.506	-37.262	**-39.913**	-22.601	-33.935	-28.553
0.015	-19.758	-36.467	-37.229	**-39.970**	-22.607	-33.861	-28.445
0.016	-19.755	-36.427	-37.194	**-40.027**	-22.613	-33.787	-28.335
0.017	-19.752	-36.387	-37.159	**-40.083**	-22.618	-33.713	-28.223
0.018	-19.749	-36.346	-37.123	**-40.138**	-22.624	-33.639	-28.110
0.019	-19.745	-36.304	-37.086	**-40.192**	-22.629	-33.565	-27.996
0.020	-19.742	-36.262	-37.047	**-40.246**	-22.633	-33.490	-27.881
0.021	-19.738	-36.219	-37.008	**-40.299**	-22.638	-33.416	-27.765
0.022	-19.733	-36.176	-36.968	**-40.351**	-22.642	-33.341	-27.647
0.023	-19.729	-36.132	-36.927	**-40.402**	-22.645	-33.266	-27.528
0.024	-19.725	-36.087	-36.885	**-40.452**	-22.649	-33.191	-27.408
0.025	-19.720	-36.042	-36.843	**-40.502**	-22.652	-33.115	-27.287
0.026	-19.715	-35.996	-36.799	**-40.551**	-22.655	-33.040	-27.165
0.027	-19.710	-35.949	-36.755	**-40.599**	-22.658	-32.964	-27.042
0.028	-19.705	-35.902	-36.709	**-40.646**	-22.660	-32.888	-26.918
0.029	-19.700	-35.855	-36.663	**-40.692**	-22.662	-32.812	-26.793
0.030	-19.694	-35.806	-36.616	**-40.737**	-22.664	-32.736	-26.668
0.031	-19.688	-35.758	-36.568	**-40.782**	-22.666	-32.660	-26.541
0.032	-19.682	-35.708	-36.519	**-40.825**	-22.667	-32.584	-26.413
0.033	-19.676	-35.658	-36.470	**-40.868**	-22.668	-32.507	-26.285
0.034	-19.670	-35.608	-36.419	**-40.910**	-22.668	-32.431	-26.156

0.035	-19.663	-35.557	-36.368	**-40.951**	-22.669	-32.354	-26.026
0.036	-19.657	-35.505	-36.316	**-40.991**	-22.669	-32.277	-25.896
0.037	-19.650	-35.453	-36.263	**-41.030**	-22.669	-32.200	-25.765
0.038	-19.643	-35.400	-36.210	**-41.068**	-22.668	-32.123	-25.633
0.039	-19.636	-35.347	-36.155	**-41.105**	-22.668	-32.046	-25.500
0.040	-19.628	-35.294	-36.100	**-41.141**	-22.667	-31.969	-25.367
0.041	-19.621	-35.239	-36.044	**-41.177**	-22.665	-31.892	-25.233
0.042	-19.613	-35.185	-35.988	**-41.211**	-22.664	-31.814	-25.099
0.043	-19.605	-35.130	-35.930	**-41.244**	-22.662	-31.737	-24.964
0.044	-19.597	-35.074	-35.872	**-41.277**	-22.660	-31.659	-24.829
0.045	-19.589	-35.018	-35.813	**-41.308**	-22.657	-31.582	-24.694
0.046	-19.580	-34.961	-35.754	**-41.339**	-22.654	-31.504	-24.557
0.047	-19.571	-34.904	-35.694	**-41.368**	-22.651	-31.426	-24.421
0.048	-19.563	-34.847	-35.633	**-41.397**	-22.648	-31.348	-24.284
0.049	-19.554	-34.789	-35.572	**-41.424**	-22.644	-31.271	-24.147
0.050	-19.544	-34.730	-35.509	**-41.450**	-22.641	-31.193	-24.009
0.051	-19.535	-34.672	-35.447	**-41.476**	-22.636	-31.115	-23.871
0.052	-19.525	-34.612	-35.383	**-41.500**	-22.632	-31.037	-23.733
0.053	-19.516	-34.553	-35.319	**-41.524**	-22.627	-30.959	-23.594
0.054	-19.506	-34.493	-35.254	**-41.546**	-22.622	-30.880	-23.456
0.055	-19.496	-34.432	-35.190	**-41.567**	-22.617	-30.802	-23.317
0.056	-19.485	-34.371	-35.145	**-41.587**	-22.611	-30.724	-23.177
0.057	-19.475	-34.310	-35.100	**-41.606**	-22.606	-30.646	-23.038
0.058	-19.464	-34.248	-35.053	**-41.625**	-22.599	-30.568	-22.898
0.059	-19.454	-34.186	-35.007	**-41.642**	-22.593	-30.489	-22.758
0.060	-19.443	-34.124	-34.959	**-41.658**	-22.586	-30.411	-22.618
0.061	-19.431	-34.061	-34.911	**-41.673**	-22.579	-30.333	-22.478
0.062	-19.420	-33.997	-34.862	**-41.686**	-22.572	-30.254	-22.338
0.063	-19.409	-33.934	-34.813	**-41.699**	-22.564	-30.176	-22.198
0.064	-19.397	-33.870	-34.763	**-41.711**	-22.557	-30.097	-22.058
0.065	-19.385	-33.806	-34.712	**-41.721**	-22.549	-30.019	-21.917
0.066	-19.373	-33.741	-34.661	**-41.731**	-22.540	-29.941	-21.777
0.067	-19.361	-33.676	-34.610	**-41.739**	-22.532	-29.862	-21.636
0.068	-19.349	-33.611	-34.557	**-41.747**	-22.523	-29.784	-21.496
0.069	-19.336	-33.545	-34.505	**-41.753**	-22.513	-29.705	-21.355
0.070	-19.323	-33.479	-34.451	**-41.758**	-22.504	-29.627	-21.215
0.071	-19.310	-33.413	-34.397	**-41.762**	-22.494	-29.548	-21.074
0.072	-19.297	-33.346	-34.343	**-41.765**	-22.484	-29.470	-20.934
0.073	-19.284	-33.280	-34.288	**-41.767**	-22.474	-29.392	-20.793
0.074	-19.271	-33.212	-34.232	**-41.768**	-22.463	-29.313	-20.653
0.075	-19.257	-33.145	-34.176	**-41.768**	-22.453	-29.235	-20.513
0.076	-19.244	-33.077	-34.120	**-41.766**	-22.441	-29.156	-20.372
0.077	-19.230	-33.009	-34.063	**-41.764**	-22.430	-29.078	-20.232

0.078	-19.216	-32.941	-34.005	**-41.760**	-22.418	-29.000	-20.092
0.079	-19.202	-32.872	-33.947	**-41.756**	-22.406	-28.921	-19.952
0.080	-19.187	-32.804	-33.888	**-41.750**	-22.394	-28.843	-19.813
0.081	-19.173	-32.735	-33.829	**-41.743**	-22.382	-28.765	-19.673
0.082	-19.158	-32.665	-33.770	**-41.735**	-22.369	-28.687	-19.534
0.083	-19.143	-32.596	-33.710	**-41.726**	-22.356	-28.608	-19.394
0.084	-19.128	-32.526	-33.649	**-41.716**	-22.343	-28.530	-19.255
0.085	-19.113	-32.456	-33.589	**-41.705**	-22.329	-28.452	-19.116
0.086	-19.098	-32.386	-33.527	**-41.693**	-22.316	-28.374	-18.977
0.087	-19.082	-32.315	-33.465	**-41.679**	-22.301	-28.296	-18.839
0.088	-19.066	-32.244	-33.403	**-41.665**	-22.287	-28.218	-18.700
0.089	-19.051	-32.173	-33.341	**-41.650**	-22.273	-28.140	-18.562
0.090	-19.035	-32.102	-33.278	**-41.633**	-22.258	-28.062	-18.424
0.091	-19.018	-32.031	-33.214	**-41.615**	-22.243	-27.984	-18.286
0.092	-19.002	-31.959	-33.150	**-41.597**	-22.227	-27.906	-18.149
0.093	-18.986	-31.888	-33.086	**-41.577**	-22.212	-27.828	-18.011
0.094	-18.969	-31.816	-33.022	**-41.556**	-22.196	-27.750	-17.874
0.095	-18.952	-31.744	-32.957	**-41.535**	-22.180	-27.673	-17.737
0.096	-18.935	-31.671	-32.891	**-41.512**	-22.163	-27.595	-17.601
0.097	-18.918	-31.599	-32.825	**-41.488**	-22.147	-27.517	-17.464
0.098	-18.901	-31.526	-32.759	**-41.463**	-22.130	-27.440	-17.328
0.099	-18.884	-31.454	-32.693	**-41.437**	-22.113	-27.362	-17.192
0.100	-18.866	-31.381	-32.626	**-41.410**	-22.095	-27.285	-17.057
0.101	-18.848	-31.307	-32.559	**-41.382**	-22.077	-27.207	-16.922
0.102	-18.830	-31.234	-32.491	**-41.353**	-22.060	-27.130	-16.787
0.103	-18.812	-31.161	-32.424	**-41.323**	-22.041	-27.053	-16.652
0.104	-18.794	-31.087	-32.355	**-41.292**	-22.023	-26.976	-16.517
0.105	-18.776	-31.014	-32.287	**-41.260**	-22.004	-26.898	-16.383
0.106	-18.758	-30.940	-32.218	**-41.227**	-21.985	-26.821	-16.249
0.107	-18.739	-30.866	-32.149	**-41.194**	-21.966	-26.744	-16.116
0.108	-18.720	-30.792	-32.080	**-41.159**	-21.947	-26.667	-15.983
0.109	-18.701	-30.717	-32.010	**-41.123**	-21.927	-26.591	-15.850
0.110	-18.682	-30.643	-31.940	**-41.086**	-21.907	-26.514	-15.717
0.111	-18.663	-30.569	-31.870	**-41.049**	-21.887	-26.437	-15.585
0.112	-18.644	-30.494	-31.799	**-41.010**	-21.867	-26.360	-15.453
0.113	-18.624	-30.419	-31.729	**-40.970**	-21.846	-26.284	-15.321
0.114	-18.604	-30.345	-31.657	**-40.930**	-21.825	-26.207	-15.190
0.115	-18.585	-30.270	-31.586	**-40.888**	-21.804	-26.131	-15.058
0.116	-18.565	-30.195	-31.515	**-40.846**	-21.783	-26.054	-14.928
0.117	-18.545	-30.120	-31.443	**-40.803**	-21.762	-25.978	-14.797
0.118	-18.524	-30.045	-31.371	**-40.759**	-21.740	-25.902	-14.667
0.119	-18.504	-29.969	-31.299	**-40.714**	-21.718	-25.826	-14.538
0.120	-18.484	-29.894	-31.226	**-40.668**	-21.696	-25.750	-14.408

0.121	-18.463	-29.819	-31.153	**-40.622**	-21.673	-25.674	-14.279
0.122	-18.442	-29.743	-31.080	**-40.574**	-21.650	-25.598	-14.150
0.123	-18.421	-29.668	-31.007	**-40.526**	-21.627	-25.522	-14.022
0.124	-18.400	-29.592	-30.934	**-40.477**	-21.604	-25.446	-13.894
0.125	-18.379	-29.516	-30.860	**-40.427**	-21.581	-25.371	-13.766
0.126	-18.358	-29.441	-30.787	**-40.376**	-21.557	-25.295	-13.639
0.127	-18.336	-29.365	-30.713	**-40.324**	-21.533	-25.220	-13.512
0.128	-18.315	-29.289	-30.638	**-40.272**	-21.509	-25.144	-13.385
0.129	-18.293	-29.213	-30.564	**-40.218**	-21.485	-25.069	-13.259
0.130	-18.271	-29.137	-30.490	**-40.165**	-21.461	-24.994	-13.133
0.131	-18.249	-29.061	-30.415	**-40.110**	-21.436	-24.919	-13.007
0.132	-18.227	-28.985	-30.340	**-40.054**	-21.411	-24.844	-12.882
0.133	-18.204	-28.909	-30.265	**-39.998**	-21.386	-24.769	-12.757
0.134	-18.182	-28.833	-30.190	**-39.941**	-21.361	-24.694	-12.632
0.135	-18.160	-28.757	-30.115	**-39.883**	-21.335	-24.619	-12.508
0.136	-18.137	-28.681	-30.039	**-39.825**	-21.309	-24.545	-12.384
0.137	-18.114	-28.605	-29.964	**-39.766**	-21.283	-24.470	-12.261
0.138	-18.091	-28.528	-29.888	**-39.706**	-21.257	-24.396	-12.138
0.139	-18.068	-28.452	-29.812	**-39.646**	-21.231	-24.321	-12.015
0.140	-18.045	-28.376	-29.736	**-39.585**	-21.204	-24.247	-11.892
0.141	-18.022	-28.299	-29.660	**-39.523**	-21.177	-24.173	-11.770
0.142	-17.998	-28.223	-29.584	**-39.460**	-21.150	-24.099	-11.649
0.143	-17.975	-28.147	-29.507	**-39.397**	-21.123	-24.025	-11.527
0.144	-17.951	-28.070	-29.431	**-39.333**	-21.096	-23.951	-11.406
0.145	-17.927	-27.994	-29.354	**-39.269**	-21.068	-23.877	-11.286
0.146	-17.903	-27.918	-29.277	**-39.204**	-21.040	-23.804	-11.165
0.147	-17.879	-27.841	-29.201	**-39.138**	-21.012	-23.730	-11.045
0.148	-17.855	-27.765	-29.124	**-39.072**	-20.984	-23.657	-10.926
0.149	-17.831	-27.689	-29.047	**-39.005**	-20.955	-23.583	-10.807
0.150	-17.806	-27.612	-28.970	**-38.938**	-20.927	-23.510	-10.688
0.151	-17.782	-27.536	-28.893	**-38.870**	-20.898	-23.437	-10.569
0.152	-17.757	-27.460	-28.815	**-38.802**	-20.869	-23.364	-10.451
0.153	-17.732	-27.383	-28.738	**-38.733**	-20.840	-23.291	-10.333
0.154	-17.707	-27.307	-28.661	**-38.663**	-20.811	-23.218	-10.216
0.155	-17.682	-27.231	-28.583	**-38.593**	-20.781	-23.145	-10.099
0.156	-17.657	-27.154	-28.506	**-38.522**	-20.751	-23.073	-9.982
0.157	-17.632	-27.078	-28.428	**-38.451**	-20.721	-23.000	-9.865
0.158	-17.606	-27.002	-28.350	**-38.380**	-20.691	-22.928	-9.749
0.159	-17.581	-26.926	-28.273	**-38.307**	-20.661	-22.855	-9.634
0.160	-17.555	-26.850	-28.195	**-38.235**	-20.630	-22.783	-9.518
0.161	-17.530	-26.773	-28.117	**-38.162**	-20.600	-22.711	-9.403
0.162	-17.504	-26.697	-28.039	**-38.088**	-20.569	-22.639	-9.289
0.163	-17.478	-26.621	-27.961	**-38.014**	-20.538	-22.567	-9.175

0.164	-17.452	-26.545	-27.883	**-37.940**	-20.507	-22.495	-9.061
0.165	-17.426	-26.469	-27.805	**-37.865**	-20.475	-22.423	-8.947
0.166	-17.399	-26.393	-27.727	**-37.790**	-20.444	-22.352	-8.834
0.167	-17.373	-26.317	-27.649	**-37.714**	-20.412	-22.280	-8.721
0.168	-17.347	-26.241	-27.571	**-37.638**	-20.380	-22.209	-8.608

Table S4: AICc values for models simulating all possible values for the year of 2005-2006 on a 0.001 increment within the range of the coordinates in the axis 1 for life history structure of the Lago Catalão fish assemblage. 100% of the best-fit model was segmented step-mean.

**Possible values**	**Null**	**Linear**	**Segmented linear-linear**	**Segmented step mean**	**Segmented linear-stable mean**	**Segmented stable mean-linear**	**Sigmoidal**
-0.135	-25.441	-27.206	-24.209	**-33.063**	-28.011	-29.464	-17.057
-0.134	-25.479	-27.253	-24.259	**-33.100**	-28.017	-29.500	-17.131
-0.133	-25.517	-27.300	-24.310	**-33.137**	-28.023	-29.534	-17.205
-0.132	-25.554	-27.347	-24.360	**-33.173**	-28.028	-29.569	-17.278
-0.131	-25.592	-27.393	-24.409	**-33.208**	-28.034	-29.603	-17.351
-0.130	-25.629	-27.439	-24.459	**-33.244**	-28.039	-29.637	-17.424
-0.129	-25.666	-27.485	-24.508	**-33.278**	-28.043	-29.670	-17.497
-0.128	-25.703	-27.531	-24.557	**-33.313**	28.048	-29.703	-17.569
-0.127	-25.739	-27.576	-24.606	**-33.346**	-28.052	29.736	-17.642
-0.126	-25.776	-27.621	-24.654	**-33.380**	-28.056	-29.769	-17.714
-0.125	-25.812	-27.666	-24.702	**-33.412**	-28.060	-29.801	-17.786
-0.124	-25.848	-27.711	-24.750	**-33.445**	-28.064	-29.832	-17.857
-0.123	-25.884	-27.755	-24.798	**-33.476**	-28.067	-29.863	-17.929
-0.122	-25.919	-27.799	-24.845	**-33.508**	-28.070	-29.894	-18.000
-0.121	-25.955	-27.843	-24.892	**-33.539**	-28.073	-29.925	-18.070
-0.120	-25.990	-27.887	-24.939	**-33.569**	-28.076	-29.955	-18.141
-0.119	-26.025	-27.930	-24.986	**-33.599**	-28.078	-29.985	-18.211
-0.118	-26.060	-27.973	-25.032	**-33.628**	-28.080	-30.014	-18.281
-0.117	-26.094	-28.015	-25.077	**-33.657**	-28.082	-30.043	-18.351
-0.116	-26.129	-28.058	-25.123	**-33.685**	-28.084	-30.072	-18.420
-0.115	-26.163	-28.100	-25.168	**-33.713**	-28.085	-30.100	-18.489
-0.114	-26.197	-28.142	-25.213	**-33.740**	-28.086	-30.127	-18.558
-0.113	-26.230	-28.183	-25.258	**-33.767**	-28.087	-30.155	-18.626
-0.112	-26.264	-28.224	-25.302	**-33.793**	-28.088	-30.182	-18.695
-0.111	-26.297	-28.265	-25.346	**-33.818**	-28.088	-30.208	-18.762
-0.110	-26.330	-28.306	-25.389	**-33.844**	-28.088	-30.235	-18.830
-0.109	-26.363	-28.346	-25.433	**-33.868**	-28.088	-30.260	-18.897
-0.108	-26.396	-28.386	-25.476	**-33.892**	-28.088	-30.286	-18.963
-0.107	-26.428	-28.425	-25.518	**-33.916**	-28.087	-30.311	-19.030
-0.106	-26.460	-28.464	-25.560	**-33.939**	-28.086	-30.335	-19.096
-0.105	-26.492	-28.503	-25.602	**-33.961**	-28.085	-30.359	-19.161
-0.104	-26.524	-28.542	-25.644	**-33.983**	-28.084	-30.383	-19.226
-0.103	-26.556	-28.580	-25.685	**-34.004**	-28.082	-30.406	-19.291

-0.102	-26.587	-28.618	-25.726	**-34.025**	-28.080	-30.429	-19.356
-0.101	-26.618	-28.656	-25.766	**-34.045**	-28.078	-30.452	-19.419
-0.100	-26.649	-28.693	-25.806	**-34.065**	-28.104	-30.474	-19.483
-0.099	-26.679	-28.730	-25.846	**-34.084**	-28.154	-30.495	-19.546
-0.098	-26.709	-28.766	-25.886	**-34.102**	-28.204	-30.516	-19.609
-0.097	-26.740	-28.802	-25.925	**-34.120**	-28.254	-30.537	-19.671
-0.096	-26.769	-28.838	-25.963	**-34.137**	-28.304	-30.557	-19.733
-0.095	-26.799	-28.874	-26.001	**-34.154**	-28.353	-30.577	-19.794
-0.094	-26.828	-28.909	-26.039	**-34.170**	-28.403	-30.597	-19.855
-0.093	-26.857	-28.944	-26.077	**-34.186**	-28.452	-30.616	-19.915
-0.092	-26.886	-28.978	-26.114	**-34.201**	-28.500	-30.634	-19.975
-0.091	-26.915	-29.012	-26.150	**-34.215**	-28.549	-30.652	-20.034
-0.090	-26.943	-29.046	-26.187	**-34.229**	-28.597	-30.670	-20.093
-0.089	-26.971	-29.079	-26.223	**-34.242**	-28.644	-30.687	-20.151
-0.088	-26.999	-29.112	-26.258	**-34.255**	-28.692	-30.704	-20.209
-0.087	-27.027	-29.144	-26.293	**-34.267**	-28.739	-30.720	-20.266
-0.086	-27.054	-29.176	-26.328	**-34.279**	-28.786	-30.736	-20.323
-0.085	-27.081	-29.208	-26.362	**-34.290**	-28.833	-30.751	-20.379
-0.084	-27.108	-29.240	-26.396	**-34.300**	-28.879	-30.766	-20.434
-0.083	-27.134	-29.270	-26.429	**-34.310**	-28.925	-30.780	-20.489
-0.082	-27.160	-29.301	-26.462	**-34.319**	-28.971	-30.794	-20.544
-0.081	-27.186	-29.331	-26.495	**-34.327**	-29.017	-30.808	-20.598
-0.080	-27.212	-29.361	-26.527	**-34.335**	-29.062	-30.821	-20.651
-0.079	-27.237	-29.390	-26.558	**-34.343**	-29.107	-30.834	-20.703
-0.078	-27.263	-29.419	-26.590	**-34.349**	-29.151	-30.846	-20.755
-0.077	-27.287	-29.448	-26.620	**-34.355**	-29.195	-30.857	-20.807
-0.076	-27.312	-29.476	-26.651	**-34.361**	-29.239	-30.869	-20.858
-0.075	-27.336	-29.504	-26.681	**-34.366**	-29.283	-30.879	-20.908
-0.074	-27.360	-29.531	-26.710	**-34.370**	-29.326	-30.890	-20.957
-0.073	-27.384	-29.558	-26.739	**-34.374**	-29.369	-30.899	-21.006
-0.072	-27.408	-29.584	-26.768	**-34.377**	-29.412	-30.909	-21.054
-0.071	-27.431	-29.610	-26.796	**-34.380**	-29.454	-30.918	-21.101
-0.070	-27.454	-29.636	-26.823	**-34.382**	-29.496	-30.926	-21.148
-0.069	-27.476	-29.661	-26.850	**-34.383**	-29.537	-30.934	-21.194
-0.068	-27.499	-29.686	-26.877	**-34.384**	-29.579	-30.941	-21.240
-0.067	-27.521	-29.710	-26.903	**-34.384**	-29.619	-30.948	-21.284
-0.066	-27.542	-29.734	-26.929	**-34.383**	-29.660	-30.955	-21.328
-0.065	-27.564	-29.758	-26.954	**-34.382**	-29.700	-30.961	-21.371
-0.064	-27.585	-29.781	-26.979	**-34.380**	-29.740	-30.966	-21.414
-0.063	-27.606	-29.803	-27.003	**-34.378**	-29.779	-30.971	-21.455
-0.062	-27.626	-29.825	-27.027	**-34.375**	-29.818	-30.976	-21.496
-0.061	-27.647	-29.847	-27.050	**-34.372**	-29.857	-30.980	-21.537
-0.060	-27.667	-29.868	-27.073	**-34.368**	-29.895	-30.983	-21.576

-0.059	-27.686	-29.889	-27.096	**-34.363**	-29.933	-30.987	-21.615
-0.058	-27.706	-29.909	-27.117	**-34.358**	-29.971	-30.989	-21.652
-0.057	-27.725	-29.929	-27.139	**-34.352**	-30.008	-30.991	-21.689
-0.056	-27.743	-29.948	-27.160	**-34.345**	-30.045	-30.993	-21.726
-0.055	-27.762	-29.967	-27.180	**-34.338**	-30.081	-30.994	-21.761
-0.054	-27.780	-29.986	-27.200	**-34.330**	-30.117	-30.995	-21.796
-0.053	-27.798	-30.004	-27.221	**-34.322**	-30.153	-30.995	-21.830
-0.052	-27.815	-30.021	-27.256	**-34.313**	-30.188	-30.995	-21.863
-0.051	-27.833	-30.038	-27.289	**-34.303**	-30.223	-30.994	-21.895
-0.050	-27.849	-30.055	-27.323	**-34.293**	-30.257	-30.993	-21.926
-0.049	-27.866	-30.071	-27.356	**-34.283**	-30.291	-31.012	-21.956
-0.048	-27.882	-30.087	-27.388	**-34.271**	-30.324	-31.030	-21.986
-0.047	-27.898	-30.102	-27.420	**-34.259**	-30.357	-31.048	-22.015
-0.046	-27.914	-30.117	-27.452	**-34.247**	-30.390	-31.065	-22.042
-0.045	-27.929	-30.131	-27.484	**-34.234**	-30.422	-31.081	-22.069
-0.044	-27.944	-30.145	-27.515	**-34.220**	-30.454	-31.097	-22.095
-0.043	-27.959	-30.158	-27.545	**-34.206**	-30.486	-31.113	-22.120
-0.042	-27.973	-30.171	-27.575	**-34.191**	-30.517	-31.128	-22.145
-0.041	-27.987	-30.183	-27.605	**-34.176**	-30.547	-31.142	-22.168
-0.040	-28.001	-30.195	-27.635	**-34.160**	-30.577	-31.156	-22.190
-0.039	-28.014	-30.206	-27.664	**-34.143**	-30.607	-31.170	-22.212
-0.038	-28.027	-30.217	-27.692	**-34.126**	-30.636	-31.183	-22.232
-0.037	-28.040	-30.228	-27.721	**-34.109**	-30.665	-31.195	-22.252
-0.036	-28.053	-30.238	-27.748	**-34.090**	-30.693	-31.207	-22.271
-0.035	-28.065	-30.247	-27.776	**-34.071**	-30.721	-31.219	-22.288
-0.034	-28.076	-30.256	-27.803	**-34.052**	-30.748	-31.230	-22.305
-0.033	-28.088	-30.264	-27.829	**-34.032**	-30.775	-31.240	-22.321
-0.032	-28.099	-30.272	-27.856	**-34.012**	-30.802	-31.250	-22.336
-0.031	-28.110	-30.280	-27.881	**-33.991**	-30.828	-31.259	-22.350
-0.030	-28.120	-30.287	-27.907	**-33.969**	-30.853	-31.268	-22.363
-0.029	-28.130	-30.293	-27.931	**-33.947**	-30.878	-31.276	-22.375
-0.028	-28.140	-30.299	-27.956	**-33.924**	-30.903	-31.284	-22.386
-0.027	-28.149	-30.305	-27.980	**-33.901**	-30.927	-31.291	-22.396
-0.026	-28.159	-30.310	-28.003	**-33.877**	-30.950	-31.298	-22.405
-0.025	-28.167	-30.314	-28.027	**-33.852**	-30.974	-31.304	-22.413
-0.024	-28.176	-30.318	-28.049	**-33.828**	-30.996	-31.309	-22.420
-0.023	-28.184	-30.322	-28.072	**-33.802**	-31.018	-31.314	-22.426
-0.022	-28.192	-30.325	-28.093	**-33.776**	-31.040	-31.319	-22.431
-0.021	-28.199	-30.327	-28.115	**-33.750**	-31.061	-31.323	-22.435
-0.020	-28.206	-30.329	-28.136	**-33.723**	-31.082	-31.326	-22.438
-0.019	-28.213	-30.330	-28.156	**-33.695**	-31.102	-31.329	-22.440
-0.018	-28.219	-30.331	-28.176	**-33.667**	-31.122	-31.332	-22.441
-0.017	-28.225	-30.332	-28.196	**-33.638**	-31.141	-31.333	-22.441

-0.016	-28.231	-30.332	-28.215	**-33.609**	-31.160	-31.335	-22.440
-0.015	-28.236	-30.331	-28.233	**-33.580**	-31.178	-31.335	-22.439
-0.014	-28.241	-30.330	-28.252	**-33.550**	-31.195	-31.336	-22.436
-0.013	-28.246	-30.329	-28.269	**-33.519**	-31.213	-31.335	-22.432
-0.012	-28.250	-30.327	-28.287	**-33.488**	-31.229	-31.334	-22.427
-0.011	-28.254	-30.324	-28.303	**-33.494**	-31.245	-31.333	-22.421
-0.010	-28.258	-30.321	-28.320	**-33.526**	-31.261	-31.331	-22.414
-0.009	-28.261	-30.318	-28.335	**-33.558**	-31.276	-31.329	-22.406
-0.008	-28.264	-30.314	-28.351	**-33.589**	-31.291	-31.325	-22.397
-0.007	-28.267	-30.309	-28.366	**-33.619**	-31.305	-31.322	-22.387
-0.006	-28.269	-30.304	-28.380	**-33.649**	-31.318	-31.318	-22.376
-0.005	-28.271	-30.299	-28.394	**-33.678**	-31.331	-31.313	-22.364
-0.004	-28.273	-30.293	-28.408	**-33.707**	-31.344	-31.308	-22.351
-0.003	-28.274	-30.286	-28.421	**-33.735**	-31.356	-31.302	-22.336
-0.002	-28.275	-30.279	-28.433	**-33.763**	-31.367	-31.296	-22.321
-0.001	-28.276	-30.272	-28.445	**-33.790**	-31.378	-31.289	-22.305
0.000	-28.276	-30.264	-28.457	**-33.816**	-31.389	-31.282	-22.288
0.001	-28.276	-30.256	-28.468	**-33.842**	-31.399	-31.274	-22.270
0.002	-28.275	-30.247	-28.478	**-33.868**	-31.408	-31.266	-22.251
0.003	-28.274	-30.237	-28.489	**-33.893**	-31.417	-31.257	-22.231
0.004	-28.273	-30.227	-28.498	**-33.917**	-31.425	-31.247	-22.210
0.005	-28.272	-30.217	-28.507	**-33.940**	-31.433	-31.238	-22.188
0.006	-28.270	-30.206	-28.516	**-33.964**	-31.440	-31.227	-22.165
0.007	-28.268	-30.195	-28.524	**-33.986**	-31.447	-31.216	-22.141
0.008	-28.265	-30.183	-28.532	**-34.008**	-31.453	-31.205	-22.116
0.009	-28.263	-30.170	-28.539	**-34.029**	-31.458	-31.192	-22.090
0.010	-28.259	-30.158	-28.546	**-34.050**	-31.463	-31.180	-22.063
0.011	-28.256	-30.144	-28.552	**-34.070**	-31.468	-31.167	-22.036
0.012	-28.252	-30.130	-28.558	**-34.089**	-31.472	-31.153	-22.007
0.013	-28.248	-30.116	-28.563	**-34.108**	-31.475	-31.139	-21.977
0.014	-28.243	-30.101	-28.567	**-34.127**	-31.478	-31.124	-21.946
0.015	-28.238	-30.086	-28.572	**-34.144**	-31.480	-31.109	-21.915
0.016	-28.233	-30.070	-28.575	**-34.161**	-31.482	-31.094	-21.882
0.017	-28.227	-30.054	-28.579	**-34.178**	-31.483	-31.077	-21.848
0.018	-28.221	-30.038	-28.581	**-34.194**	-31.484	-31.061	-21.814
0.019	-28.215	-30.021	-28.584	**-34.209**	-31.484	-31.043	-21.779
0.020	-28.209	-30.003	-28.585	**-34.223**	-31.484	-31.026	-21.742
0.021	-28.202	-29.985	-28.587	**-34.237**	-31.483	-31.008	-21.705
0.022	-28.194	-29.966	-28.587	**-34.251**	-31.482	-30.989	-21.667
0.023	-28.187	-29.948	-28.588	**-34.263**	-31.480	-30.970	-21.628
0.024	-28.179	-29.928	-28.587	**-34.275**	-31.477	-30.950	-21.588
0.025	-28.170	-29.908	-28.587	**-34.287**	-31.474	-30.930	-21.547
0.026	-28.162	-29.888	-28.585	**-34.298**	-31.470	-30.909	-21.506

0.027	-28.153	-29.867	-28.584	**-34.308**	-31.466	-30.888	-21.463
0.028	-28.143	-29.846	-28.581	**-34.317**	-31.462	-30.866	-21.420
0.029	-28.134	-29.824	-28.579	**-34.326**	-31.456	-30.844	-21.375
0.030	-28.124	-29.802	-28.575	**-34.334**	-31.451	-30.821	-21.330
0.031	-28.114	-29.779	-28.572	**-34.342**	-31.444	-30.798	-21.284
0.032	-28.103	-29.756	-28.567	**-34.349**	-31.438	-30.775	-21.237
0.033	-28.092	-29.733	-28.563	**-34.355**	-31.430	-30.750	-21.190
0.034	-28.081	-29.709	-28.558	**-34.361**	-31.422	-30.726	-21.141
0.035	-28.069	-29.685	-28.552	**-34.366**	-31.414	-30.701	-21.092
0.036	-28.057	-29.660	-28.546	**-34.371**	-31.405	-30.675	-21.042
0.037	-28.045	-29.635	-28.539	**-34.374**	-31.395	-30.649	-20.991
0.038	-28.032	-29.609	-28.532	**-34.378**	-31.385	-30.623	-20.940
0.039	-28.019	-29.583	-28.524	**-34.380**	-31.375	-30.596	-20.887
0.040	-28.006	-29.557	-28.516	**-34.382**	-31.364	-30.569	-20.834
0.041	-27.992	-29.530	-28.507	**-34.383**	-31.352	-30.541	-20.780
0.042	-27.978	-29.502	-28.498	**-34.384**	-31.340	-30.513	-20.725
0.043	-27.964	-29.475	-28.489	**-34.384**	-31.327	-30.484	-20.670
0.044	-27.950	-29.447	-28.478	**-34.383**	-31.314	-30.455	-20.614
0.045	-27.935	-29.418	-28.468	**-34.382**	-31.300	-30.425	-20.557
0.046	-27.920	-29.389	-28.457	**-34.380**	-31.286	-30.395	-20.499
0.047	-27.904	-29.360	-28.445	**-34.377**	-31.271	-30.365	-20.441
0.048	-27.888	-29.330	-28.433	**-34.374**	-31.256	-30.334	-20.382
0.049	-27.872	-29.300	-28.421	**-34.370**	-31.240	-30.302	-20.322
0.050	-27.856	-29.269	-28.408	**-34.365**	-31.224	-30.271	-20.262
0.051	-27.839	-29.238	-28.394	**-34.360**	-31.207	-30.238	-20.201
0.052	-27.822	-29.207	-28.380	**-34.354**	-31.190	-30.206	-20.139
0.053	-27.804	-29.175	-28.366	**-34.347**	-31.172	-30.173	-20.076
0.054	-27.787	-29.143	-28.351	**-34.340**	-31.154	-30.139	-20.013
0.055	-27.769	-29.110	-28.335	**-34.332**	-31.135	-30.106	-19.949
0.056	-27.750	-29.077	-28.320	**-34.324**	-31.115	-30.071	-19.885
0.057	-27.732	-29.044	-28.303	**-34.315**	-31.095	-30.037	-19.820
0.058	-27.713	-29.010	-28.286	**-34.305**	-31.075	-30.001	-19.755
0.059	-27.693	-28.976	-28.269	**-34.295**	-31.054	-29.966	-19.688
0.060	-27.674	-28.942	-28.252	**-34.284**	-31.033	-29.930	-19.622
0.061	-27.654	-28.907	-28.233	**-34.272**	-31.011	-29.894	-19.554
0.062	-27.634	-28.872	-28.215	**-34.260**	-30.989	-29.857	-19.486
0.063	-27.613	-28.837	-28.196	**-34.247**	-30.966	-29.820	-19.418
0.064	-27.593	-28.801	-28.176	**-34.234**	-30.943	-29.783	-19.349
0.065	-27.572	-28.765	-28.156	**-34.220**	-30.919	-29.745	-19.279
0.066	-27.550	-28.728	-28.136	**-34.205**	-30.895	-29.707	-19.209
0.067	-27.529	-28.691	-28.115	**-34.190**	-30.870	-29.668	-19.138
0.068	-27.507	-28.654	-28.093	**-34.174**	-30.845	-29.629	-19.067
0.069	-27.484	-28.616	-28.071	**-34.157**	-30.819	-29.590	-18.995

0.070	-27.462	-28.578	-28.049	**-34.140**	-30.793	-29.550	-18.923
0.071	-27.439	-28.540	-28.027	**-34.122**	-30.766	-29.510	-18.850
0.072	-27.416	-28.502	-28.003	**-34.103**	-30.739	-29.470	-18.777
0.073	-27.393	-28.463	-27.980	**-34.084**	-30.712	-29.429	-18.703
0.074	-27.369	-28.423	-27.956	**-34.065**	-30.684	-29.388	-18.629
0.075	-27.345	-28.384	-27.931	**-34.045**	-30.655	-29.346	-18.554
0.076	-27.321	-28.344	-27.907	**-34.024**	-30.626	-29.305	-18.479
0.077	-27.296	-28.304	-27.881	**-34.002**	-30.597	-29.262	-18.403
0.078	-27.272	-28.263	-27.856	**-33.980**	-30.567	-29.220	-18.327
0.079	-27.247	-28.222	-27.829	**-33.958**	-30.537	-29.177	-18.251
0.080	-27.221	-28.181	-27.803	**-33.934**	-30.506	-29.134	-18.174
0.081	-27.196	-28.140	-27.776	**-33.911**	-30.475	-29.091	-18.097
0.082	-27.170	-28.098	-27.748	**-33.886**	-30.444	-29.047	-18.019
0.083	-27.144	-28.056	-27.721	**-33.861**	-30.412	-29.003	-17.941
0.084	-27.117	-28.013	-27.692	**-33.836**	-30.379	-28.958	-17.863
0.085	-27.091	-27.971	-27.664	**-33.810**	-30.347	-28.914	-17.784
0.086	-27.064	-27.928	-27.635	**-33.783**	-30.313	-28.869	-17.705
0.087	-27.036	-27.885	-27.605	**-33.756**	-30.280	-28.823	-17.625
0.088	-27.009	-27.841	-27.575	**-33.728**	-30.246	-28.778	-17.545
0.089	-26.981	-27.797	-27.545	**-33.700**	-30.211	-28.732	-17.465
0.090	-26.953	-27.753	-27.515	**-33.671**	-30.176	-28.687	-17.384
0.091	-26.925	-27.709	-27.483	**-33.641**	-30.141	-28.643	-17.303
0.092	-26.897	-27.664	-27.452	**-33.611**	-30.105	-28.599	-17.222
0.093	-26.868	-27.619	-27.420	**-33.581**	-30.069	-28.555	-17.140
0.094	-26.839	-27.574	-27.388	**-33.550**	-30.033	-28.510	-17.058
0.095	-26.810	-27.529	-27.355	**-33.518**	-29.996	-28.465	-16.976
0.096	-26.780	-27.483	-27.323	**-33.486**	-29.958	-28.419	-16.894
0.097	-26.750	-27.437	-27.289	**-33.453**	-29.921	-28.374	-16.811
0.098	-26.720	-27.391	-27.256	**-33.420**	-29.883	-28.328	-16.728
0.099	-26.690	-27.344	-27.221	**-33.386**	-29.844	-28.282	-16.644
0.100	-26.660	-27.298	-27.187	**-33.352**	-29.805	-28.235	-16.561
0.101	-26.629	-27.251	-27.152	**-33.317**	-29.766	-28.189	-16.477
0.102	-26.598	-27.204	-27.117	**-33.282**	-29.727	-28.142	-16.393
0.103	-26.567	-27.156	-27.082	**-33.246**	-29.687	-28.094	-16.308
0.104	-26.536	-27.108	-27.046	**-33.210**	-29.647	-28.047	-16.224
0.105	-26.504	-27.061	-27.009	**-33.173**	-29.606	-27.999	-16.139
0.106	-26.472	-27.012	-26.973	**-33.135**	-29.565	-27.951	-16.054
0.107	-26.440	-26.964	-26.936	**-33.098**	-29.524	-27.903	-15.969
0.108	-26.408	-26.916	-26.899	**-33.059**	-29.482	-27.854	-15.883
0.109	-26.375	-26.867	-26.861	**-33.021**	-29.440	-27.805	-15.797
0.110	-26.342	-26.818	-26.823	**-32.981**	-29.398	-27.756	-15.712
0.111	-26.309	-26.768	-26.785	**-32.942**	-29.355	-27.707	-15.625
0.112	-26.276	-26.719	-26.746	**-32.902**	-29.312	-27.657	-15.539

0.113	-26.243	-26.669	-26.707	**-32.861**	-29.269	-27.607	-15.453
0.114	-26.209	-26.620	-26.668	**-32.820**	-29.225	-27.557	-15.366
0.115	-26.175	-26.569	-26.628	**-32.778**	-29.181	-27.507	-15.279
0.116	-26.141	-26.519	-26.589	**-32.736**	-29.136	-27.456	-15.192
0.117	-26.107	-26.469	-26.548	**-32.694**	-29.092	-27.406	-15.105
0.118	-26.072	-26.418	-26.508	**-32.651**	-29.047	-27.355	-15.018
0.119	-26.037	-26.367	-26.467	**-32.608**	-29.002	-27.303	-14.930
0.120	-26.003	-26.316	-26.426	**-32.564**	-28.956	-27.252	-14.843
0.121	-25.967	-26.265	-26.384	**-32.520**	-28.910	-27.200	-14.755
0.122	-25.932	-26.213	-26.343	**-32.475**	-28.864	-27.149	-14.667
0.123	-25.897	-26.162	-26.301	**-32.430**	-28.817	-27.097	-14.579
0.124	-25.861	-26.110	-26.258	**-32.385**	-28.771	-27.044	-14.491
0.125	-25.825	-26.058	-26.216	**-32.339**	-28.724	-26.992	-14.403
0.126	-25.789	-26.006	-26.173	**-32.293**	-28.676	-26.939	-14.315
0.127	-25.752	-25.954	-26.129	**-32.246**	-28.629	-26.886	-14.226
0.128	-25.716	-25.901	-26.086	**-32.199**	-28.581	-26.833	-14.138
0.129	-25.679	-25.849	-26.042	**-32.151**	-28.533	-26.780	-14.049
0.130	-25.642	-25.796	-25.998	**-32.104**	-28.484	-26.727	-13.960
0.131	-25.605	-25.743	-25.954	**-32.055**	-28.435	-26.673	-13.872
0.132	-25.568	-25.690	-25.909	**-32.007**	-28.386	-26.619	-13.783
0.133	-25.531	-25.636	-25.864	**-31.958**	-28.337	-26.565	-13.694
0.134	-25.493	-25.583	-25.819	**-31.908**	-28.288	-26.511	-13.605
0.135	-25.455	-25.529	-25.774	**-31.859**	-28.238	-26.457	-13.516
0.136	-25.417	-25.476	-25.728	**-31.809**	-28.188	-26.402	-13.427
0.137	-25.379	-25.422	-25.690	**-31.758**	-28.138	-26.348	-13.337
0.138	-25.341	-25.368	-25.657	**-31.708**	-28.087	-26.293	-13.248
0.139	-25.302	-25.314	-25.624	**-31.656**	-28.036	-26.238	-13.159
0.140	-25.263	-25.259	-25.591	**-31.605**	-27.985	-26.183	-13.070
0.141	-25.225	-25.205	-25.557	**-31.553**	-27.934	-26.128	-12.980
0.142	-25.186	-25.150	-25.524	**-31.501**	-27.883	-26.072	-12.891
0.143	-25.146	-25.095	-25.490	**-31.449**	-27.831	-26.017	-12.801
0.144	-25.107	-25.041	-25.455	**-31.396**	-27.779	-25.961	-12.712
0.145	-25.067	-24.986	-25.421	**-31.343**	-27.727	-25.905	-12.622
0.146	-25.028	-24.931	-25.386	**-31.289**	-27.675	-25.849	-12.533
0.147	-24.988	-24.875	-25.351	**-31.235**	-27.622	-25.793	-12.443
0.148	-24.948	-24.820	-25.316	**-31.181**	-27.569	-25.736	-12.354

Table S5: AICc values for models simulating all possible values for the year of 2005-2006 on a 0.001 increment within the range of the coordinates in the axis 1 for vertical consumer trophic levels structure of the Lago Catalão fish assemblage. 74% of the best-fit model was segmented linear- linear, and 26% of the best-fit model was segmented step-mean.

**Possible values**	**Null**	**Linear**	**Segmented linear-linear**	**Segmented step mean**	**Segmented linear-stable mean**	**Segmented stable mean-linear**	**Sigmoidal**
-0.098	-25.566	-24.226	-25.436	**-30.356**	-25.458	-25.299	-13.071
-0.097	-25.593	-24.254	-25.508	**-30.371**	-25.460	-25.319	-13.097
-0.096	-25.621	-24.282	-25.579	**-30.386**	-25.461	-25.338	-13.121
-0.095	-25.648	-24.309	-25.650	**-30.401**	-25.463	-25.357	-13.146
-0.094	-25.675	-24.337	-25.721	**-30.415**	-25.464	-25.376	-13.170
-0.093	-25.701	-24.363	-25.793	**-30.429**	-25.465	-25.394	-13.194
0.092	-25.728	-24.390	-25.864	**-30.442**	-25.465	-25.412	-13.217
-0.091	-25.754	-24.416	-25.935	**-30.455**	-25.466	-25.430	-13.241
-0.090	-25.780	-24.442	26.006	**30.467**	-25.466	-25.448	-13.263
-0.089	-25.806	-24.468	-26.077	**-30.479**	-25.466	-25.465	-13.286
-0.088	-25.831	-24.494	-26.148	**-30.491**	-25.466	-25.482	-13.308
-0.087	-25.856	-24.519	-26.219	**-30.502**	-25.465	-25.499	-13.330
-0.086	-25.881	-24.544	-26.290	**-30.513**	-25.465	-25.515	-13.351
-0.085	-25.906	-24.568	-26.360	**-30.523**	-25.464	-25.531	-13.372
-0.084	-25.931	-24.593	-26.431	**-30.533**	-25.463	-25.547	-13.393
-0.083	-25.955	-24.617	-26.502	**-30.542**	-25.462	-25.562	-13.413
-0.082	-25.979	-24.640	-26.572	**-30.551**	-25.461	-25.577	-13.433
-0.081	-26.003	-24.664	-26.643	**-30.559**	-25.459	-25.592	-13.453
-0.080	-26.026	-24.687	-26.713	**-30.567**	-25.457	-25.606	-13.472
-0.079	-26.050	-24.710	-26.783	**-30.574**	-25.455	-25.620	-13.491
-0.078	-26.073	-24.733	-26.854	**-30.581**	-25.453	-25.634	-13.510
-0.077	-26.095	-24.755	-26.924	**-30.588**	-25.451	-25.648	-13.528
-0.076	-26.118	-24.777	-26.994	**-30.594**	-25.448	-25.661	-13.546
-0.075	-26.140	-24.798	-27.064	**-30.600**	-25.445	-25.674	-13.563
-0.074	-26.162	-24.820	-27.134	**-30.605**	-25.442	-25.686	-13.580
-0.073	-26.184	-24.841	-27.203	**-30.610**	-25.439	-25.699	-13.597
-0.072	-26.205	-24.862	-27.273	**-30.614**	-25.435	-25.711	-13.613
-0.071	-26.226	-24.882	-27.343	**-30.618**	-25.432	-25.722	-13.629
-0.070	-26.247	-24.902	-27.412	**-30.621**	-25.428	-25.734	-13.645
-0.069	-26.268	-24.922	-27.481	**-30.624**	-25.424	-25.744	-13.660
-0.068	-26.288	-24.942	-27.551	**-30.626**	-25.420	-25.755	-13.675
-0.067	-26.308	-24.961	-27.620	**-30.628**	-25.415	-25.765	-13.690

-0.066	-26.328	-24.980	-27.689	**-30.630**	-25.411	-25.775	-13.704
-0.065	-26.348	-24.998	-27.758	**-30.631**	-25.406	-25.785	-13.718
-0.064	-26.367	-25.017	-27.826	**-30.631**	-25.401	-25.794	-13.731
-0.063	-26.386	-25.035	-27.895	**-30.631**	-25.396	-25.803	-13.744
-0.062	-26.405	-25.052	-27.963	**-30.631**	-25.390	-25.812	-13.757
-0.061	-26.423	-25.069	-28.032	**-30.630**	-25.384	-25.821	-13.769
-0.060	-26.442	-25.086	-28.100	**-30.629**	-25.379	-25.829	-13.781
-0.059	-26.459	-25.103	-28.168	**-30.627**	-25.373	-25.836	-13.793
-0.058	-26.477	-25.119	-28.236	**-30.625**	-25.366	-25.844	-13.804
-0.057	-26.494	-25.135	-28.303	**-30.622**	-25.366	-25.851	-13.815
-0.056	-26.512	-25.151	-28.371	**-30.619**	-25.391	-25.857	-13.825
-0.055	-26.528	-25.167	-28.438	**-30.615**	-25.417	-25.864	-13.835
-0.054	-26.545	-25.182	-28.506	**-30.611**	-25.441	-25.870	-13.845
-0.053	-26.561	-25.196	-28.573	**-30.607**	-25.466	-25.876	-13.854
-0.052	-26.577	-25.211	-28.639	**-30.602**	-25.490	-25.881	-13.863
-0.051	-26.593	-25.225	-28.706	**-30.596**	-25.514	-25.886	-13.871
-0.050	-26.608	-25.238	-28.773	**-30.590**	-25.537	-25.891	-13.879
-0.049	-26.623	-25.252	-28.839	**-30.584**	-25.560	-25.895	-13.887
-0.048	-26.638	-25.265	-28.905	**-30.577**	-25.583	-25.899	-13.894
-0.047	-26.652	-25.277	-28.971	**-30.570**	-25.606	-25.903	-13.901
-0.046	-26.667	-25.290	-29.037	**-30.562**	-25.628	-25.906	-13.908
-0.045	-26.680	-25.302	-29.102	**-30.554**	-25.650	-25.910	-13.914
-0.044	-26.694	-25.314	-29.167	**-30.545**	-25.671	-25.912	-13.920
-0.043	-26.707	-25.325	-29.233	**-30.536**	-25.693	-25.915	-13.925
-0.042	-26.720	-25.336	-29.297	**-30.527**	-25.714	-25.917	-13.930
-0.041	-26.733	-25.347	-29.362	**-30.517**	-25.734	-25.919	-13.935
-0.040	-26.746	-25.357	-29.427	**-30.506**	-25.755	-25.920	-13.939
-0.039	-26.758	-25.367	-29.491	**-30.495**	-25.775	-25.921	-13.943
-0.038	-26.770	-25.377	-29.555	**-30.484**	-25.794	-25.922	-13.946
-0.037	-26.781	-25.386	-29.618	**-30.472**	-25.814	-25.922	-13.950
-0.036	-26.792	-25.395	-29.682	**-30.460**	-25.833	-25.922	-13.952
-0.035	-26.803	-25.403	-29.745	**-30.447**	-25.851	-25.922	-13.955
-0.034	-26.814	-25.412	-29.808	**-30.434**	-25.869	-25.921	-13.956
-0.033	-26.824	-25.420	-29.871	**-30.420**	-25.887	-25.920	-13.958
-0.032	-26.834	-25.427	-29.933	**-30.406**	-25.905	-25.919	-13.959
-0.031	-26.844	-25.434	-29.995	**-30.392**	-25.922	-25.917	-13.960
-0.030	-26.854	-25.441	-30.057	**-30.377**	-25.939	-25.916	-13.960
-0.029	-26.863	-25.448	-30.119	**-30.362**	-25.956	-25.924	-13.960
-0.028	-26.871	-25.454	-30.180	**-30.346**	-25.972	-25.931	-13.960
-0.027	-26.880	-25.460	-30.241	**-30.330**	-25.988	-25.938	-13.959
-0.026	-26.888	-25.465	-30.302	**-30.313**	-26.004	-25.945	-13.958
-0.025	-26.896	-25.470	**-30.362**	-30.296	-26.019	-25.952	-13.956
-0.024	-26.904	-25.475	**-30.422**	-30.278	-26.034	-25.958	-13.954

-0.023	-26.911	-25.480	**-30.482**	-30.261	-26.048	-25.964	-13.952
-0.022	-26.918	-25.484	**-30.542**	-30.242	-26.063	-25.969	-13.949
-0.021	-26.925	-25.487	**-30.601**	-30.223	-26.076	-25.974	-13.946
-0.020	-26.931	-25.491	**-30.660**	-30.204	-26.090	-25.979	-13.943
-0.019	-26.937	-25.494	**-30.718**	-30.185	-26.103	-25.983	-13.939
-0.018	-26.943	-25.496	**-30.777**	-30.164	-26.116	-25.987	-13.934
-0.017	-26.948	-25.499	**-30.834**	-30.144	-26.128	-25.991	-13.930
-0.016	-26.953	-25.501	**-30.892**	-30.123	-26.140	-25.994	-13.925
-0.015	-26.958	-25.502	**-30.949**	-30.102	-26.152	-25.997	-13.919
-0.014	-26.963	-25.504	**-31.006**	-30.080	-26.163	-25.999	-13.914
-0.013	-26.967	-25.504	**-31.062**	-30.058	-26.174	-26.001	-13.907
-0.012	-26.971	-25.505	**-31.118**	-30.067	-26.185	-26.003	-13.901
-0.011	-26.974	-25.505	**-31.174**	-30.088	-26.195	-26.004	-13.894
-0.010	-26.977	-25.505	**-31.229**	-30.109	-26.205	-26.005	-13.886
-0.009	-26.980	-25.504	**-31.284**	-30.130	-26.215	-26.006	-13.879
-0.008	-26.983	-25.504	**-31.339**	-30.150	-26.224	-26.006	-13.871
-0.007	-26.985	-25.502	**-31.393**	-30.169	-26.233	-26.006	-13.862
-0.006	-26.987	-25.501	**-31.447**	-30.188	-26.241	-26.006	-13.853
-0.005	-26.989	-25.499	**-31.500**	-30.207	-26.249	-26.005	-13.844
-0.004	-26.990	-25.497	**-31.553**	-30.225	-26.257	-26.004	-13.835
-0.003	-26.991	-25.494	**-31.606**	-30.243	-26.264	-26.002	-13.825
-0.002	-26.992	-25.491	**-31.658**	-30.260	-26.271	-26.000	-13.814
-0.001	-26.993	-25.488	**-31.710**	-30.277	-26.278	-25.998	-13.804
0.000	-26.993	-25.484	**-31.761**	-30.293	-26.284	-25.995	-13.792
0.001	-26.992	-25.480	**-31.812**	-30.309	-26.290	-25.992	-13.781
0.002	-26.992	-25.475	**-31.862**	-30.325	-26.295	-25.989	-13.769
0.003	-26.991	-25.471	**-31.912**	-30.340	-26.300	-25.985	-13.757
0.004	-26.990	-25.466	**-31.961**	-30.354	-26.305	-25.981	-13.744
0.005	-26.989	-25.460	**-32.010**	-30.368	-26.310	-25.976	-13.732
0.006	-26.987	-25.454	**-32.059**	-30.381	-26.314	-25.971	-13.718
0.007	-26.985	-25.448	**-32.107**	-30.394	-26.317	-25.966	-13.705
0.008	-26.982	-25.442	**-32.155**	-30.407	-26.321	-25.960	-13.691
0.009	-26.980	-25.435	**-32.202**	-30.419	-26.323	-25.954	-13.676
0.010	-26.977	-25.428	**-32.248**	-30.431	-26.326	-25.948	-13.662
0.011	-26.973	-25.420	**-32.295**	-30.442	-26.328	-25.941	-13.646
0.012	-26.970	-25.412	**-32.340**	-30.452	-26.330	-25.934	-13.631
0.013	-26.966	-25.404	**-32.385**	-30.462	-26.331	-25.927	-13.615
0.014	-26.961	-25.395	**-32.430**	-30.474	-26.332	-25.919	-13.599
0.015	-26.957	-25.386	**-32.474**	-30.486	-26.333	-25.911	-13.583
0.016	-26.952	-25.377	**-32.518**	-30.498	-26.333	-25.902	-13.566
0.017	-26.947	-25.368	**-32.561**	-30.509	-26.333	-25.893	-13.548
0.018	-26.941	-25.358	**-32.603**	-30.519	-26.333	-25.884	-13.531
0.019	-26.935	-25.347	**-32.645**	-30.530	-26.332	-25.875	-13.513

0.020	-26.929	-25.337	**-32.687**	-30.539	-26.331	-25.865	-13.495
0.021	-26.923	-25.326	**-32.728**	-30.549	-26.329	-25.854	-13.476
0.022	-26.916	-25.314	**-32.768**	-30.557	-26.327	-25.844	-13.457
0.023	-26.909	-25.303	**-32.808**	-30.566	-26.325	-25.833	-13.438
0.024	-26.902	-25.291	**-32.847**	-30.573	-26.323	-25.821	-13.418
0.025	-26.894	-25.278	**-32.886**	-30.581	-26.319	-25.809	-13.398
0.026	-26.886	-25.266	**-32.924**	-30.587	-26.316	-25.797	-13.378
0.027	-26.878	-25.253	**-32.962**	-30.594	-26.312	-25.785	-13.358
0.028	-26.869	-25.239	**-32.999**	-30.600	-26.308	-25.772	-13.337
0.029	-26.860	-25.226	**-33.035**	-30.605	-26.304	-25.759	-13.315
0.030	-26.851	-25.212	**-33.071**	-30.610	-26.299	-25.745	-13.294
0.031	-26.841	-25.197	**-33.106**	-30.614	-26.293	-25.731	-13.272
0.032	-26.832	-25.183	**-33.141**	-30.618	-26.288	-25.717	-13.249
0.033	-26.821	-25.168	**-33.175**	-30.621	-26.282	-25.703	-13.227
0.034	-26.811	-25.152	**-33.208**	-30.624	-26.275	-25.688	-13.204
0.035	-26.800	-25.137	**-33.241**	-30.627	-26.269	-25.673	-13.181
0.036	-26.789	-25.121	**-33.273**	-30.628	-26.262	-25.657	-13.157
0.037	-26.778	-25.104	**-33.305**	-30.630	-26.254	-25.641	-13.133
0.038	-26.766	-25.088	**-33.336**	-30.631	-26.246	-25.625	-13.109
0.039	-26.754	-25.071	**-33.366**	-30.631	-26.238	-25.608	-13.085
0.040	-26.742	-25.053	**-33.396**	-30.631	-26.229	-25.591	-13.060
0.041	-26.730	-25.036	**-33.425**	-30.631	-26.221	-25.574	-13.035
0.042	-26.717	-25.018	**-33.453**	-30.629	-26.211	-25.556	-13.009
0.043	-26.704	-25.000	**-33.481**	-30.628	-26.202	-25.538	-12.983
0.044	-26.690	-24.981	**-33.508**	-30.626	-26.192	-25.520	-12.957
0.045	-26.677	-24.962	**-33.534**	-30.623	-26.181	-25.502	-12.931
0.046	-26.663	-24.943	**-33.560**	-30.620	-26.170	-25.483	-12.904
0.047	-26.648	-24.923	**-33.585**	-30.617	-26.159	-25.463	-12.878
0.048	-26.634	-24.904	**-33.610**	-30.613	-26.148	-25.444	-12.850
0.049	-26.619	-24.883	**-33.633**	-30.608	-26.136	-25.424	-12.823
0.050	-26.604	-24.863	**-33.656**	-30.603	-26.124	-25.404	-12.795
0.051	-26.588	-24.842	**-33.679**	-30.598	-26.111	-25.383	-12.767
0.052	-26.573	-24.821	**-33.701**	-30.592	-26.098	-25.362	-12.738
0.053	-26.557	-24.800	**-33.722**	-30.585	-26.085	-25.341	-12.710
0.054	-26.540	-24.778	**-33.742**	-30.578	-26.072	-25.319	-12.681
0.055	-26.524	-24.756	**-33.762**	-30.571	-26.058	-25.297	-12.652
0.056	-26.507	-24.734	**-33.781**	-30.563	-26.043	-25.275	-12.622
0.057	-26.490	-24.711	**-33.799**	-30.555	-26.029	-25.253	-12.592
0.058	-26.472	-24.689	**-33.817**	-30.546	-26.014	-25.230	-12.562
0.059	-26.455	-24.665	**-33.833**	-30.536	-25.998	-25.207	-12.532
0.060	-26.437	-24.642	**-33.850**	-30.526	-25.983	-25.183	-12.501
0.061	-26.418	-24.618	**-33.865**	-30.516	-25.967	-25.160	-12.470
0.062	-26.400	-24.594	**-33.880**	-30.505	-25.950	-25.136	-12.439

0.063	-26.381	-24.570	**-33.894**	-30.494	-25.933	-25.111	-12.408
0.064	-26.362	-24.545	**-33.907**	-30.482	-25.916	-25.087	-12.376
0.065	-26.342	-24.520	**-33.920**	-30.470	-25.899	-25.062	-12.344
0.066	-26.323	-24.495	**-33.932**	-30.457	-25.881	-25.037	-12.312
0.067	-26.303	-24.470	**-33.943**	-30.444	-25.863	-25.011	-12.279
0.068	-26.283	-24.444	**-33.954**	-30.430	-25.845	-24.985	-12.247
0.069	-26.262	-24.418	**-33.963**	-30.416	-25.826	-24.959	-12.214
0.070	-26.241	-24.392	**-33.972**	-30.401	-25.807	-24.933	-12.180
0.071	-26.220	-24.365	**-33.981**	-30.386	-25.787	-24.906	-12.147
0.072	-26.199	-24.338	**-33.988**	-30.371	-25.768	-24.879	-12.113
0.073	-26.178	-24.311	**-33.995**	-30.354	-25.748	-24.852	-12.079
0.074	-26.156	-24.284	**-34.001**	-30.338	-25.727	-24.824	-12.045
0.075	-26.134	-24.256	**-34.007**	-30.321	-25.706	-24.796	-12.011
0.076	-26.112	-24.228	**-34.011**	-30.304	-25.685	-24.768	-11.976
0.077	-26.089	-24.200	**-34.015**	-30.286	-25.664	-24.740	-11.941
0.078	-26.066	-24.172	**-34.019**	-30.267	-25.642	-24.711	-11.906
0.079	-26.043	-24.143	**-34.021**	-30.249	-25.620	-24.682	-11.870
0.080	-26.020	-24.114	**-34.023**	-30.229	-25.598	-24.653	-11.835
0.081	-25.996	-24.085	**-34.024**	-30.210	-25.575	-24.623	-11.799
0.082	-25.972	-24.055	**-34.024**	-30.189	-25.552	-24.593	-11.763
0.083	-25.948	-24.025	**-34.024**	-30.169	-25.529	-24.563	-11.727
0.084	-25.924	-23.995	**-34.022**	-30.148	-25.505	-24.533	-11.690
0.085	-25.899	-23.965	**-34.020**	-30.126	-25.481	-24.502	-11.653
0.086	-25.875	-23.935	**-34.018**	-30.104	-25.457	-24.471	-11.616
0.087	-25.849	-23.904	**-34.014**	-30.082	-25.433	-24.440	-11.579
0.088	-25.824	-23.873	**-34.010**	-30.059	-25.408	-24.409	-11.542
0.089	-25.799	-23.842	**-34.006**	-30.036	-25.383	-24.377	-11.504
0.090	-25.773	-23.810	**-34.000**	-30.012	-25.357	-24.345	-11.466
0.091	-25.747	-23.778	**-33.994**	-29.988	-25.331	-24.313	-11.428
0.092	-25.720	-23.746	**-33.987**	-29.963	-25.305	-24.281	-11.390
0.093	-25.694	-23.714	**-33.979**	-29.938	-25.279	-24.249	-11.352
0.094	-25.667	-23.682	**-33.970**	-29.913	-25.252	-24.216	-11.313
0.095	-25.640	-23.649	**-33.961**	-29.887	-25.225	-24.184	-11.274
0.096	-25.613	-23.616	**-33.951**	-29.861	-25.198	-24.151	-11.235
0.097	-25.586	-23.583	**-33.941**	-29.834	-25.171	-24.118	-11.196
0.098	-25.558	-23.549	**-33.929**	-29.807	-25.143	-24.085	-11.157
0.099	-25.530	-23.516	**-33.917**	-29.780	-25.115	-24.052	-11.117
0.100	-25.502	-23.482	**-33.904**	-29.752	-25.086	-24.018	-11.077
0.101	-25.473	-23.448	**-33.891**	-29.723	-25.058	-23.984	-11.037
0.102	-25.445	-23.414	**-33.877**	-29.695	-25.029	-23.950	-10.997
0.103	-25.416	-23.379	**-33.862**	-29.666	-25.000	-23.916	-10.957
0.104	-25.387	-23.344	**-33.846**	-29.636	-24.970	-23.881	-10.916
0.105	-25.358	-23.309	**-33.830**	-29.606	-24.940	-23.846	-10.876

0.106	-25.328	-23.274	**-33.813**	-29.576	-24.910	-23.811	-10.835
0.107	-25.299	-23.239	**-33.795**	-29.545	-24.880	-23.776	-10.794
0.108	-25.269	-23.203	**-33.776**	-29.514	-24.849	-23.740	-10.753
0.109	-25.239	-23.168	**-33.757**	-29.483	-24.819	-23.704	-10.711
0.110	-25.208	-23.132	**-33.737**	-29.451	-24.787	-23.668	-10.670
0.111	-25.178	-23.095	**-33.717**	-29.419	-24.756	-23.632	-10.628
0.112	-25.147	-23.059	**-33.696**	-29.386	-24.724	-23.596	-10.586
0.113	-25.116	-23.022	**-33.674**	-29.353	-24.692	-23.559	-10.544
0.114	-25.085	-22.986	**-33.651**	-29.320	-24.660	-23.522	-10.502
0.115	-25.054	-22.949	**-33.628**	-29.286	-24.628	-23.485	-10.460
0.116	-25.022	-22.911	**-33.604**	-29.252	-24.595	-23.448	-10.417
0.117	-24.990	-22.874	**-33.580**	-29.217	-24.562	-23.411	-10.374
0.118	-24.958	-22.836	**-33.554**	-29.183	-24.529	-23.373	-10.332
0.119	-24.926	-22.799	**-33.528**	-29.147	-24.496	-23.335	-10.289
0.120	-24.894	-22.761	**-33.502**	-29.112	-24.462	-23.297	-10.245
0.121	-24.861	-22.722	**-33.475**	-29.076	-24.428	-23.259	-10.202
0.122	-24.829	-22.684	**-33.447**	-29.040	-24.403	-23.220	-10.159
0.123	-24.796	-22.646	**-33.418**	-29.003	-24.385	-23.181	-10.115
0.124	-24.763	-22.607	**-33.389**	-28.966	-24.366	-23.143	-10.071
0.125	-24.729	-22.568	**-33.359**	-28.929	-24.346	-23.104	-10.028
0.126	-24.696	-22.529	**-33.329**	-28.891	-24.327	-23.064	-9.984
0.127	-24.662	-22.490	**-33.298**	-28.854	-24.307	-23.025	-9.939
0.128	-24.628	-22.450	**-33.266**	-28.815	-24.287	-22.985	-9.895
0.129	-24.594	-22.411	**-33.234**	-28.777	-24.267	-22.945	-9.851
0.130	-24.560	-22.371	**-33.201**	-28.738	-24.247	-22.905	-9.806
0.131	-24.526	-22.331	**-33.167**	-28.699	-24.226	-22.865	-9.762
0.132	-24.491	-22.291	**-33.133**	-28.659	-24.205	-22.825	-9.717
0.133	-24.456	-22.251	**-33.098**	-28.619	-24.184	-22.784	-9.672
0.134	-24.421	-22.210	**-33.063**	-28.579	-24.163	-22.744	-9.627
0.135	-24.386	-22.170	**-33.027**	-28.538	-24.141	-22.703	-9.582
0.136	-24.351	-22.129	**-32.990**	-28.498	-24.120	-22.662	-9.537
0.137	-24.315	-22.088	**-32.953**	-28.457	-24.098	-22.620	-9.491
0.138	-24.280	-22.047	**-32.916**	-28.415	-24.075	-22.579	-9.446
0.139	-24.244	-22.006	**-32.877**	-28.374	-24.053	-22.537	-9.400
0.140	-24.208	-21.964	**-32.839**	-28.332	-24.030	-22.496	-9.354
0.141	-24.172	-21.923	**-32.799**	-28.289	-24.008	-22.454	-9.308
0.142	-24.136	-21.881	**-32.759**	-28.247	-23.985	-22.412	-9.262
0.143	-24.099	-21.839	**-32.719**	-28.204	-23.961	-22.369	-9.216
0.144	-24.063	-21.797	**-32.678**	-28.161	-23.938	-22.327	-9.170
0.145	-24.026	-21.755	**-32.636**	-28.117	-23.914	-22.285	-9.124
0.146	-23.989	-21.713	**-32.594**	-28.074	-23.890	-22.242	-9.078
0.147	-23.952	-21.671	**-32.551**	-28.030	-23.866	-22.199	-9.031
0.148	-23.915	-21.628	**-32.508**	-27.985	-23.842	-22.156	-8.984

0.149	-23.877	-21.585	**-32.464**	-27.941	-23.818	-22.113	-8.938
0.150	-23.840	-21.543	**-32.420**	-27.896	-23.793	-22.070	-8.891
0.151	-23.802	-21.500	**-32.375**	-27.851	-23.768	-22.026	-8.844
0.152	-23.764	-21.457	**-32.330**	-27.806	-23.743	-21.983	-8.797
0.153	-23.726	-21.413	**-32.284**	-27.760	-23.718	-21.939	-8.750
0.154	-23.688	-21.370	**-32.238**	-27.714	-23.692	-21.895	-8.703
0.155	-23.650	-21.327	**-32.191**	-27.668	-23.667	-21.851	-9.989
0.156	-23.611	-21.283	**-32.144**	-27.622	-23.641	-21.807	-9.938
0.157	-23.573	-21.239	**-32.096**	-27.575	-23.615	-21.763	-9.887
0.158	-23.534	-21.195	**-32.048**	-27.529	-23.588	-21.718	-9.836
0.159	-23.495	-21.151	**-31.999**	-27.482	-23.562	-21.674	-9.784
0.160	-23.456	-21.107	**-31.950**	-27.434	-23.535	-21.629	-9.733
0.161	-23.417	-21.063	**-31.901**	-27.387	-23.508	-21.584	-9.681
0.162	-23.378	-21.019	**-31.851**	-27.339	-23.481	-21.540	-9.630
0.163	-23.339	-20.974	**-31.800**	-27.291	-23.454	-21.495	-9.578
0.164	-23.299	-20.930	**-31.749**	-27.243	-23.427	-21.449	-9.526
0.165	-23.260	-20.885	**-31.698**	-27.195	-23.399	-21.404	-9.474
0.166	-23.220	-20.840	**-31.646**	-27.146	-23.371	-21.359	-9.421
0.167	-23.180	-20.795	**-31.594**	-27.097	-23.343	-21.313	-9.369
0.168	-23.140	-20.750	**-31.541**	-27.048	-23.315	-21.268	-9.317
0.169	-23.100	-20.705	**-31.488**	-26.999	-23.287	-21.222	-9.264
0.170	-23.060	-20.660	**-31.435**	-26.950	-23.258	-21.176	-9.211
0.171	-23.019	-20.615	**-31.381**	-26.900	-23.230	-21.130	-9.158
0.172	-22.979	-20.569	**-31.327**	-26.850	-23.201	-21.084	-9.105
0.173	-22.938	-20.524	**-31.272**	-26.800	-23.172	-21.038	-9.052
0.174	-22.897	-20.478	**-31.217**	-26.750	-23.143	-20.991	-8.998
0.175	-22.857	-20.433	**-31.162**	-26.699	-23.113	-20.945	-8.945
0.176	-22.816	-20.387	**-31.106**	-26.649	-23.084	-20.899	-8.891
0.177	-22.775	-20.341	**-31.050**	-26.598	-23.054	-20.852	-8.837
0.178	-22.733	-20.295	**-30.993**	-26.547	-23.024	-20.805	-8.783
0.179	-22.692	-20.249	**-30.936**	-26.496	-22.994	-20.759	-8.729

Table S6: Repeated-measures ANOVA testing the main effects of before and after 2005 drought on abundance (CPUE) for each functional group.

		Mean CPUE before	Mean CPUE after	SS	df	MS	F	P
Equilibrium Small	Drought	0.067	0.023	0.0059	1	0.0256	35.751	< 0.001
	Error			0.0018	11	0.00065		
Equilibrium Large	Drought	0.024	0.008	0.00079	1	0.000079	17.77	0.001
	Error			0.00045	11	0.000045		
Intermediate Small	Drought	0.261	0.118	0.0634	1	0.0634	36.20	< 0.001
	Error			0.0192	11	0.0175		
Periodic Small	Drought	0.874	0.638	0.1835	1	0.1835	34.93	< 0.001
	Error			0.3111	11	0.0282		
Periodic Large	Drought	0.207	0.097	0.0385	1	0.0385	6.48	0.027
	Error			0.0121	11	0.0011		
Consumer Trophic Level 1	Drought	0.364	0.314	0.0076	1	0.0076	0.843	0.378
	Error			0.0994	11	0.0090		
Consumer Trophic Level 1.5	Drought	0.511	0.279	0.1642	1	0.1642	16.77	0.001
	Error			0.1076	11	0.0097		
Consumer Trophic Level 2	Drought	0.271	0.129	0.0616	1	0.0616	53.69	< 0.001
	Error			0.0126	11	0.0011		
Consumer Trophic Level 3	Drought	0.284	0.151	0.0542	1	0.0542	42.48	< 0.001
	Error			0.0140	11	0.0012		

Table S7: Repeated-measures ANOVA testing the main effects of after 2005 drought and after 2010 drought on fish abundance (CPUE) for each functional group.

		Mean 2005	Mean 2010	SS	df	MS	F	P
Equilibrium Small	Drought	0.028	0.019	0.0002	1	0.0002	0.9568	0.3658
	Error			0.0011	6	0.0002		
Equilibrium Large	Drought	0.008	0.008	0.0000	1	0.0000	0.0002	0.9883
	Error			0.0001	6	0.0000		
Intermediate Small	Drought	0.119	0.116	0.0000	1	0.0000	0.0081	0.9310
	Error			0.0166	6	0.0028		
Periodic Small	Drought	0.668	0.592	0.0006	1	0.0006	1.7852	0.2300
	Error			0.0020	6	0.0003		
Periodic Large	Drought	0.104	0.086	0.0115	1	0.0115	0.3052	0.6006
	Error			0.2266	6	0.0378		
Consumer Trophic Level 1	Drought	0.362	0.267	0.0181	1	0.0181	1.6265	0.2493
	Error			0.0669	6	0.0111		
Consumer Trophic Level 1.5	Drought	0.280	0.275	0.0000	1	0.0000	0.0004	0.9846
	Error			0.0826	6	0.0138		
Consumer Trophic Level 2	Drought	0.130	0.128	0.0000	1	0.0000	0.0184	0.8966
	Error			0.0053	6	0.0009		
Consumer Trophic Level 3	Drought	0.155	0.148	0.0001	1	0.0001	0.0816	0.7847
	Error			0.0061	6	0.0010		

References Supporting Information – sequence continue from the main text

59. Espinoza-Villar, J. C. *et al*., Contrasting regional discharge evolutions in the Amazon basin (1974–2004). *J Hydrol* **375**, 297–311 (2009).

60. Brito, J. G., Alves, L. F. & Espirito Santo, H. M. V. Seasonal and spatial variations in limnological conditions of a floodplain lake (Lake Catalão) connected to both the Solimões and Negro rivers, Central Amazonia. *Acta Amazonica* **44**, 121–134 (2014).

61. Almeida, F. F. & Melo, S. Considerações limnológicas sobre um lago da planície de inundação amazônica (lago Catalão – Estado do Amazonas, Brasil). *Acta Sci Biol Sci* **31**, 387–395 (2009).

62. Aprile, F. & Darwich, A. J. Nutrients and water-forest interactions in an Amazon floodplain lake: an ecological approach. *Acta Limnol. Bras.* **25**, 169–182 (2013).

63. Caraballo, P., Forsberg, B. R., Almeida, F. F. de & Leite, R. G. Diel patterns of temperature, conductivity and dissolved oxygen in an Amazon floodplain lake: description of a *friagem* phenomenon. *Acta Limnol. Bras.* **26**, 318–331 (2014).

64. Chellappa, S., Câmara M.R., Chellappa, N. T., Beveridge, M. C. M., Huntingford, F. A. Reproductive ecology of a neotropical cichlid fish, *Cichla monoculus* (Osteichthyes: Cichlidae). *Braz. J. Biol.* **63**, 17-26 (2003).

65. Mérona, B. de & Rankin-de-Mérona, J. Food resource partitioning in a fish community of the central Amazon floodplain. *Neotrop. Ichthyol.* **2**, 75-84 (2004).

66. Lowrya, D. *et al.* Aerial and aquatic feeding in the silver arawana, *Osteoglossum bicirrhosum*. *Environ. Biol. Fishes* **73**, 453-462 (2005).

67. Favero, J.M. del, Pompeu, P dos S., Prado-Valladares, A.C. Aspectos reprodutivos de duas espécies de ciclídeos na Reserva de Desenvolvimento Sustentável Amanã, Amazonas, Brasil. *Rev. Bras. Zoociên.* **12**, 117-124 (2010).

68. Leibel, W.S. Heckel's Thread-finned Acara. *Freshwater and Marine Aquarium* 7-5 (1984).

69. Fontenele, O. Contribuição para o conhecimento da biologia do apaiari, *Astronotus ocellatus* (Spix) (Pisces, Cichlidae) em cativeiro: aparelho de reprodução, hábitos de desova e prolificidade. *Rev. Bras. Biol.* **2**, 467-484 (1951).

70. Favero, J.M. del, Pompeu, P. dos S., Prado-Valladares, A.C. Biologia reprodutiva de *Heros efasciatus* Heckel, 1840 (Pisces, Cichlidae) na Reserva de Desenvolvimento Sustentável Amanã-AM, visando seu manejo sustentável. *Acta Amazonica* **40**, 373-380 (2010).

71. Röpke, C. P., Ferreira, E. F., Zuanon, J. Seasonal changes in the use of feeding resources by fish in stands of aquatic macrophytes in an Amazonian floodplain. Brazil. *Environ. Biol. Fishes* **97**, 401-414 (2014).

72. Ortega-Salas, A. A., Cortés, G.I. & Reyes-Bustamante, H. Fecundity, growth, and survival of the angelfish *Pterophyllum scalare* (Perciformes: Cichlidae) under laboratory conditions. *Rev. Biol. Trop.* **57**, 741-747 (2009).

73. Prado, C. P. A, Gomiero, L. M. & Froehlich, O. Spawning and parental care in *Hoplias malabaricus* (Teleostei, Characiformes, Erythrinidae) in the southern Pantanal, Brazil. *Braz. J. Biol.* **66**, 697-702 (2006).

74. Winemiller, K. O. Feeding and reproductive biology of the currito, *Hoplosternum littorale*, in the Venezuelan llanos with comments on the possible function of the enlarged male pectoral spines. *Environ. Biol. Fishes* **20**, 219-227 (1987).

75. Hostache, G. & Mol, J. H. Reproductive biology of the neotropical armoured catfish *Hoplosternum littorale* (Siluriformes – Callichthyidae): a synthesis stressing the role of floating bubble nest. *Aquat. Livin. Resour.* **11**, 173- 185 (1998).

76. Correa, S. B. & Winemiller, K. O. Niche partitioning among frugivorous fishes in response to fluctuating resources in the Amazonian floodplain forest. *Ecology* **95**, 10-224 (2014).

77. Queiroz, H.L., Sobanski, M.B. & Magurran, A. E. Reproductive strategies of Red-bellied Piranha (*Pygocentrus nattereri* Kner, 1858) in the white waters of the Mamirauá flooded forest, central Brazilian Amazon. *Environ. Biol. Fishes* **89**, 11-19 (2010).

78. Agostinho, C. S. Reproductive aspects of piranhas *Serrasalmus spilopleura* and *Serrasalmus marginatus* into the upper Paraná River, Brazil. *Braz. J. Biol.* **63**, 1-6 (2003).

79. Peressin, A., Gonçalves, C. da S. & Braga, F. M. de S. Reproductive strategies of two Curimatidae species in a Mogi Guaçu impoundment, upper Paraná River basin, São Paulo, Brazil. *Neotrop. Ichthyol.* **10**, 847-854 (2012).

80. Vazzoler, A. E. A. M. & Menezes, N. A. Síntese dos conhecimentos sobre o comportamento reprodutivo dos Characiformes da América do Sul (Teleostei, Ostariophysi). *Rev. Bras. Biol.* **52**, 627-640 (1992).

81. Zhang, J., Takita, T. & Zhang, C. Reproductive biology of *Ilisha elongata* (Teleostei: Pristigasteridae) in Ariake Sound, Japan: Implications for estuarine fish conservation in Asia. *Estuar. Coast. and Shelf Sci*. **81**,105-113 (2009).

82. Alves, M. I. M. & Filho, A. A. S. Aspectos da reprodução do jaraqui escama fina, *Semaprochilodus taeniurus* (Vallenciennes, 1881). *Ciên. Agron*. **23**, 123-137 (1992).

83. Nascimento, M. M. do, Nascimento, W. S., Chellappa, N. T. & Chellappa, S. Biologia reprodutiva do curimatã comum, *Prochilodus brevis* (Characiformes: Prochilodontidae) no açude Marechal Dutra, Rio Grande do Norte, Brasil. *Biot. Amaz.* **2**, 31-43 (2012).

84. Neuberger, A. L., Marques, E.E., Agostinho, C. S. & Oliveira, R. J. de Reproductive biology of *Rhaphiodon vulpinus* (Ostariophysi: Cynodontidae) in the Tocantins River Basin, Brazil. *Neotrop. Ichthyol*. **5**, 479-484 (2007).

85. Gomeiro, L. M. & Braga, F. M. S. Reproduction of Pirapitinga do Sul (*Brycon opalinus* Cuvier, 1819) in the Parque Estadual da Serra do Mar-Núcleo Santa Virgínia, São Paulo, Brazil. *Braz. J. Biol.* **67**, 541-549 (2007).

86. Vieira, E. F., Isaac, V.J. & Fabré, N. N. Biologia reprodutiva do tambaqui, *Colossoma macropomum* Cuvier, 1818 (Teleostei, Serrasalmidae), no baixo Amazonas, Brasil. *Acta Amazonica* **29**, 625-638 (1999).

87. Araújo-Lima, C. A. R. M., Forsberg, B. R., Victória, R. L. & Martinelli, L. A. Energy sources for detritivorous fishes in the Amazon. *Science* **234**, 256-1258 (1986).

88. Forsberg, B. R., Araújo-Lima, C. A. R. M., Martinelli, L. A., Victoria, R. L. & Bonassi, J. A. Autotrophic carbon sources for fish of the Central Amazon. *Ecology* **74**, 643-652 (1993).
